# Six-year epidemiological dynamics of human respiratory syncytial virus infections in children in central China (2019-2024): pandemic suppression, 2023 resurgence, and immune debt effect

**DOI:** 10.3389/fcimb.2025.1691957

**Published:** 2026-01-13

**Authors:** Zhiyi Xia, Xue Li, Adong Shen, Igor Mokrousov, Pengbo Guo, Yaodong Zhang

**Affiliations:** 1 Henan Children’s Hospital Zhengzhou Children’s Hospital, Zhengzhou, China; 2 Henan International Joint Laboratory of Children’s Infectious Diseases, Children’s Hospital Affiliated to Zhengzhou University, Henan Children’s Hospital Zhengzhou Children’s Hospital, Zhengzhou, China; 3 Laboratory of Molecular Epidemiology and Evolutionary Genetics, Saint Petersburg Pasteur Institute, Saint-Petersburg, Russia; 4 Children’s Medical Research Institute of Henan Academy of Medical Sciences, Zhengzhou, China

**Keywords:** human respiratory syncytial virus (HRSV), children, immune debt, pandemic suppression, post-pandemic resurgence

## Abstract

**Background:**

Human respiratory syncytial virus (HRSV) is a leading cause of acute respiratory infections in children. COVID-19 NPIs significantly suppressed HRSV transmission. This study analyzed six-year epidemiological dynamics of pediatric HRSV infections in Henan Province, China, focusing on NPI suppression effects, the 2023 resurgence, and “Immune debt” impact.

**Methods:**

We retrospectively collected respiratory specimens from 80,920 children with acute respiratory diseases at Henan Children’s Hospital (2019-2024). HRSV was detected using RT-qPCR. Positivity rates were analyzed by year, season, and age group.

**Results:**

During 2019–2024, HRSV positivity fluctuated markedly: 14.65% (2019), 16.34% (2021), 3.27% (2022 under strict NPIs), 21.47% (2023 post-NPIs), and 6.80% (2024). Interrupted time-series analysis indicated that NPI lifting in 2023 was associated with a significant surge in infection risk (OR = 668.77, 95% CI: 47.03–9509.28). Seasonal patterns shifted substantially, with the characteristic winter peak replaced by an off-season spring outbreak in April 2023 (57.41%). Multivariable logistic regression identified age as the strongest predictor, with infants <1 year having the highest risk (aOR = 9.02, 95% CI: 8.31–9.79) and a 4.91-fold higher positivity rate than school-aged children (22.98% vs. 4.68%; 95% CI: 4.59–5.25; P < 0.001).

**Conclusions:**

NPIs dramatically affected HRSV epidemiology. The intense post-suppression rebound strongly supports the “Immune debt” theory—accumulation of susceptible children driving resurgence. Establishing year-round, multi-pathogen surveillance systems is crucial for post-pandemic public health challenges.

## Introduction

Human respiratory syncytial virus (HRSV) is a single-strand, negative-sense RNA virus with a single serotype (Griffiths et al., 2017). Highly contagious, HRSV is primarily transmitted through respiratory droplets or contact with contaminated surfaces. It causes a spectrum of acute respiratory illnesses, ranging from upper respiratory tract infections (e.g., nasal congestion, rhinorrhea, cough, wheezing) to severe lower respiratory tract diseases such as pneumonia and bronchiolitis (Shi et al; Borchers et al., 2013). HRSV infections occur worldwide, with prevalence varying by geography, temperature, and humidity. Epidemics typically peak during winter and spring, starting in November/December, reaching their height in February, and subsiding by April, lasting approximately 24 weeks (Zhang et al., 2015; Chadha et al., 2020). As a major infectious disease threat, particularly in children, HRSV is a leading global cause of morbidity and mortality among children under 5 years (GBD 2013 Mortality and Causes of Death Collaborators, 2025; Li et al., 2022). It poses a serious threat to children’s health, with the highest risk of severe disease and prolonged hospitalization in those under 3 years old. HRSV infection can be complicated by co-infection with other viruses or bacteria, including Streptococcus pneumoniae, Staphylococcus aureus, Haemophilus influenzae, and Mycoplasma pneumoniae. Co-infections are associated with increased disease severity, potentially leading to respiratory failure, multi-organ dysfunction, a higher risk of death, and an elevated disease burden (Rodriguez et al., 2014; Meskill and O’Bryant, 2020; Lodi et al., 2024).

The COVID-19 pandemic profoundly disrupted the global transmission dynamics of respiratory viruses (Olsen et al., 2020; Perofsky et al., 2024). Non-pharmaceutical interventions (NPIs), such as masking, school closures, and travel restrictions, suppressed the circulation of multiple pathogens, including Mycoplasma pneumoniae (MP) and enveloped viruses like influenza and HRSV, worldwide during 2020-2022 (Burki, 2021; Meyer and Beeton, 2023; Principi et al., 2023; Chen et al., 2024; Qiu et al., 2024; Yue et al., 2025). However, systematic analyses of HRSV epidemiology that encompass both the pandemic suppression phase and the post-pandemic resurgence phase remain critically lacking for Central China. Notably, this suppression of pathogen circulation has been hypothesized to induce an “immune debt”—defined as the accumulation of immunologically naive individuals (particularly children) due to reduced exposure to pathogens during NPIs, which in turn fuels intense post-intervention infection resurgences. The theoretical framework of immune debt involves two key layers: at the individual level, limited pathogen exposure impairs the development of adaptive immunity to specific pathogens; at the population level, the expanded pool of susceptible individuals amplifies transmission once NPIs are lifted (Cohen et al., 2021; Cohen et al., 2023).

Henan Province, a core region in Central China, stands as one of the most populous regions in the country, with a permanent population of approximately 97.85 million—including a substantial pediatric population of around 29 million children (China Census 2024; https://chinacensus.org/province). This large and densely populated setting, coupled with its sizeable child demographic, underscores the significance of robust epidemiological research based on extensive datasets from the region. Here, we present a 6-year surveillance study (2019-2024) on HRSV in Central China, providing novel evidence of epidemiological disruptions driven by NPIs. Our findings reveal that the unprecedented resurgence of HRSV in 2023 aligns strongly with the “Immune debt” hypothesis, offering important insights for integrating respiratory pathogen surveillance into regional public health preparedness frameworks.

## Materials and methods

### Ethics approval

This study was approved by the Ethics Committee of Henan Children’s Hospital (Approval number: 2023-K-L023), in compliance with international ethical standards, including the 1975 Helsinki Declaration (revised 2000). Written informed consent was obtained from all patients or their parents/legal guardians.

### Study population and clinical criteria

This retrospective study included children (aged 0–18 years) who presented to the hospital with symptoms of acute respiratory tract infection (ARI) and subsequently underwent physician-ordered testing for HRSV From January 1, 2019 to December 31, 2024.

### Inclusion criteria

1. Children aged 0–18 years, Divided into 4 groups, <1, 1-3 ([1,3)), 3–6 [3,6), 6–18 [6,18).
2. Clinical presentation with symptoms suggestive of ARI, including but not limited to: cough, fever, rhinorrhea, wheezing, respiratory distress, or sore throat.
3. Availability of a valid RT-qPCR test result for HRSV from a respiratory specimen (nasopharyngeal swab, sputum, or bronchoalveolar lavage fluid).

### Exclusion criteria

1. Patients with incomplete medical records or missing demographic information.
2. Samples that were improperly collected, transported, or deemed invalid per laboratory quality control protocols (e.g., internal control failure).
3. Patients with known significant congenital malformations, primary immunodeficiency, malignant tumors, or those receiving immunosuppressive therapy, as these conditions could atypically alter the risk or presentation of respiratory viral infections.

### Testing indication

Testing for HRSV was initiated at the discretion of the treating physician based on clinical assessment. The decision to test was typically driven by the presence of ARI symptoms, particularly in young children and infants, or in cases where the clinical presentation suggested bronchiolitis or pneumonia.

### Detection methods

Samples were tested using a commercial amplification kit (Shengxiang, Cat. No. 20213400256, China) and nucleic acid extraction reagent (Shengxiang, Cat. No. 20210488, China) per the manufacturer’s instructions. Real-time quantitative PCR (RT-qPCR) was performed on the Hongshi SLAN-96P Fully Automatic Medical PCR Analysis System with TaqMan probes. The internal control was the human housekeeping gene RNase P (RPP40, Gene ID: 10799), and the target gene was the HRSV M gene (Gene ID: 37607640). This assay participated in the external quality assessment (EQA) for inter-laboratory comparison organized by Jinshui District General Hospital, Zhengzhou, China. It was a single-target assay for HRSV, with no consideration of co-infecting pathogens. Result interpretation: Samples with a clear S-shaped amplification curve and cycle threshold (Ct) < 40 were positive; those with no amplification curve or Ct > 40 were negative. For negative samples, the internal control must have Ct < 40. Assays were invalid if the internal control had Ct > 40 or no amplification—after identifying and excluding causes, such samples were re-collected and retested. Invalid retests were discarded and not included in the total count. The lower limit of detection (LOD) was 500 copies/mL.

### Interrupted time-series and seasonal modeling

To quantitatively assess the impact of key policy interventions on HRSV epidemiology, we conducted an interrupted time-series analysis using a negative binomial generalized linear model. Following the initial outbreak, China implemented a dynamic zero-COVID strategy characterized by localized and short-term control measures. A significant shift in policy intensity occurred in March 2022 (T1), when widespread Omicron transmission prompted stricter, larger-scale, and prolonged cross-city lockdowns in Henan Province, representing a substantial escalation of NPIs that led to a sharp decline in HRSV testing volume. This intensive intervention period was followed by a major policy reversal in January 2023 (T2), when the nationwide lifting of strict NPIs marked the end of large-scale containment efforts. Subsequently, with widespread clinical and public acceptance of RT-qPCR technology, testing volume surged dramatically in 2024. These two points—T1 (March 2022, representing strict NPIs implementation) and T2 (January 2023, representing NPIs lifting)—were defined as critical intervention points. The model included terms for baseline trend, immediate level changes, and trend changes following each intervention. Robust standard errors (HC3) accounted for heteroscedasticity, and counterfactual analysis estimated expected HRSV positivity rates absent interventions.

Complementary analyses included classical seasonal decomposition (additive model, period=12 months) to examine trend, seasonal, and residual components, and harmonic regression incorporating sinusoidal terms (cos[2πt/12], sin[2πt/12]) to quantify seasonal amplitude and phase shifts across intervention periods.

### Multivariate logistic regression

Multivariable logistic regression assessed independent risk factors for HRSV infection. The model included age groups (reference: 6–18 years), gender, patient type (inpatient/outpatient), seasonal harmonics (month_sin, month_cos), and policy periods (T1: strict NPIs; T2_2023, T2_2024: post-NPIs years). Results are presented as adjusted odds ratios (aOR) with 95% confidence intervals. Model fit was evaluated using pseudo R² and AIC.

### Statistical analysis and visualization​

Statistical analyses were performed using SPSS 21.0 and Python 3.9 (scipy.stats, statsmodels, matplotlib). A two-tailed P < 0.05 was significant, with adjustments for multiple comparisons as specified.

### Descriptive statistics

HRSV positivity rates (with 95% CIs, Wilson score method) and case counts were stratified by year (2019–2024), season, age group (<1 year [further subdivided], 1–3 years, 3–6 years, 6–18 years), and gender.

### Group comparison & trend analysis

Group comparisons: Pearson’s chi-square test (Fisher’s exact test if >20% cells had expected frequency <5) compared positivity rates across independent groups (e.g., gender, policy phases).

#### Trend analysis

Cochran-Armitage trend test evaluated monotonic positivity trends (e.g., age-related declines, temporal trends).

### Regression modeling

Multivariate logistic regression: Identified independent HRSV infection predictors (age, gender, season, policy phase, patient type), with model fit assessed via pseudo-R² and AIC.

#### Negative binomial count regression

Analyzed monthly positive counts (offset by log test volume) to quantify policy effects and seasonal fluctuations.

### Multiple comparison correction

Bonferroni correction adjusted P-values for multiple tests (e.g., **Figure 7**, multivariate logistic regression subgroup comparisons) to control Type I errors.

## Results

### Study population and testing volume: HRSV testing data from Henan Children’s Hospital (2019-2024)

In this study, data were collected from children tested for HRSV at Henan Children’s Hospital. After excluding cases with incomplete clinical data, a total of 80,920 children were included in the final analysis (**Table 1**). Testing volumes varied across the study period, with the lowest number recorded in 2020 (3,954 cases) and the highest in 2024 (39,793 cases). The annual testing volumes for other years were as follows: 9,284 in 2019, 10,759 in 2021, 4,192 in 2022, and 12,938 in 2023.

**Table 1:** Basic information of HRSV detection from 2019 to 2024.

Characteristic (n=80920)		2019 (n=9284)	2020 (n=3954)	2021 (n=10759)	2022 (n=4192)	2023 (n=12938)	2024 (n=39793)
Total positive count		1360	624	1758	137	2778	2704
Total positive rate(%)		14.65	15.78	16.34	3.27	21.47	6.8
95% CI (%)		13.94-15.38	14.68-16.95	15.65-17.05	2.77-3.85	20.77-22.19	6.55-7.05
male	positive count	803	381	1070	87	1610	1586
	total count	5484	2381	6478	2487	7378	22678
	positive rate(%)	14.64	16	16.52	3.5	21.82	6.99
	95% CI (%)	13.73-15.6	14.58-17.53	15.63-17.44	2.84-4.3	20.89-22.78	6.67-7.33
female	positive Female	557	243	688	50	1168	1118
	total Female	3800	1573	4281	1705	5560	17115
	positive rate(%)	14.66	15.45	16.07	2.93	21.01	6.53
	95% CI (%)	13.57-15.82	13.75-17.32	15-17.2	2.23-3.85	19.96-22.1	6.17-6.91
Sex difference	Chi2	0	0.18	0.34	0.85	1.2	3.2
	p_value	1	0.67	0.56	0.36	0.27	0.07

### Age-specific vulnerability: Infants under 1 year are at greatest risk, with a 4.91-fold higher positivity rate than school-aged children (6–18 years)

**Figure 1:**
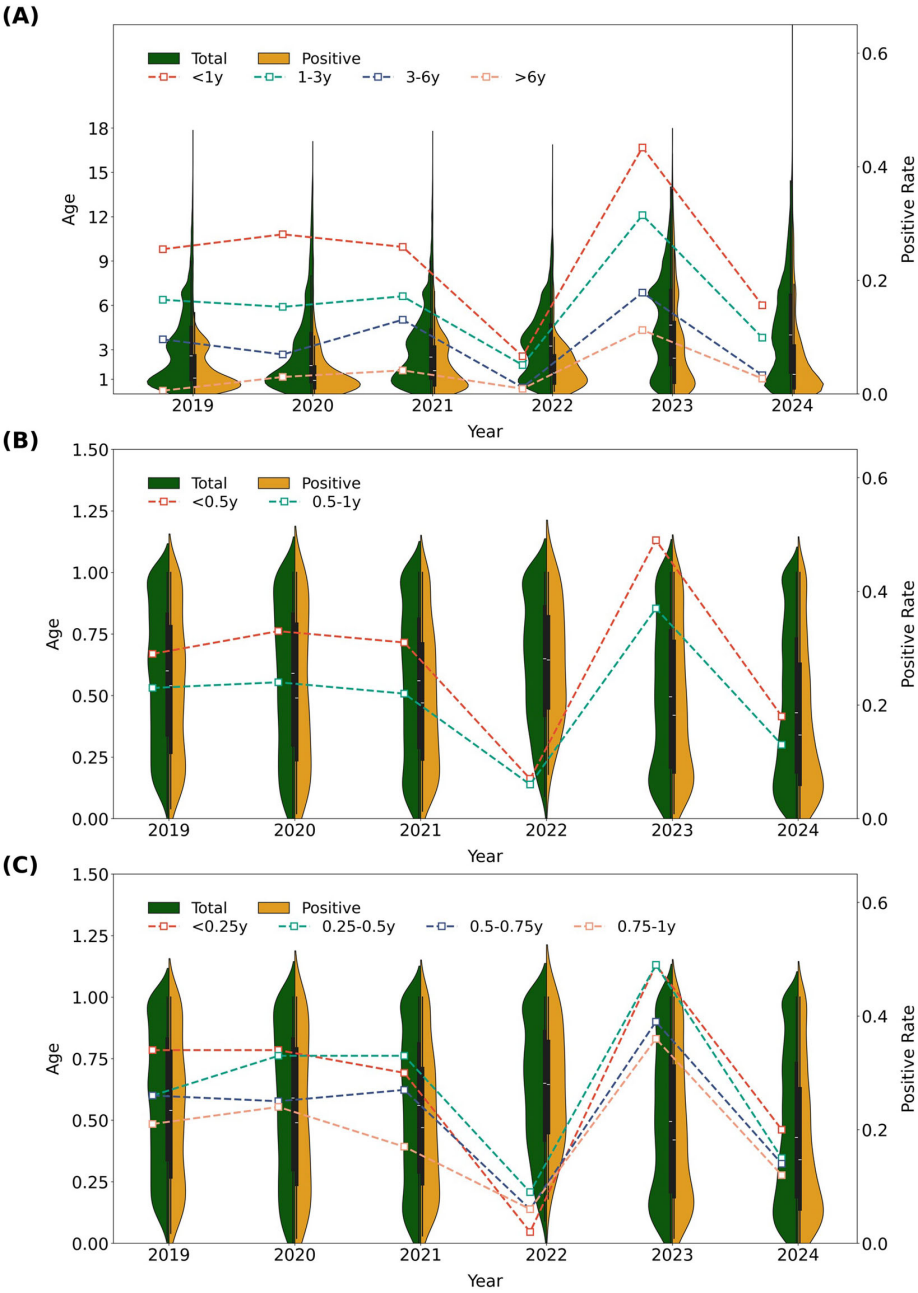
HRSV positivity 2019–2024: overall trends, sex differences, and infant subgroup analyses. **(A)** Age group comparison: <1, 1-3, 3-6, and 6–18 years. **(B)** Infant subgroup comparison: <0.5 vs. 0.5–1 year. **(C)** Infant subgroup comparison: 0-0.25, 0.25-0.5, 0.5-0.75, and 0.75–1 year.

Age-stratified analysis revealed striking differences in HRSV susceptibility: across all years, positivity rates showed a monotonic decrease with age (Cochran_Armitage_P <0.001), peaking in the <1 year group, which had a 4.91-fold higher positivity rate than school-aged children (6–18 years) (P<0.001) (**Figure 1A**, **Table 2**):

**Table 2:** Number of HRSV positive cases and positivity rate by age group from 2019 to 2024.

Year	Age group	Positive cases	Total number	Positivity rate	Cochran_ armitage_P	Slope	RR (<1 group vs 6–18 group,95%CI)
2019	<1	614	2357	26.05%	<0.001	-8.348	46.89 (23.42-93.89) P<0.001
	1-3	468	2764	16.93%			
	3-6	270	2723	9.92%			
	6-18	8	1440	0.56%			
2020	<1	334	1173	28.47%	<0.001	-8.536	9.82 (6.09-15.82) P<0.001
	1-3	206	1286	16.02%			
	3-6	67	909	7.37%			
	6-18	17	586	2.90%			
2021	<1	637	2375	26.82%	<0.001	-7.24	6.65 (5.13-8.64) P<0.001
	1-3	618	3581	17.26%			
	3-6	445	3364	13.23%			
	6-18	58	1439	4.03%			
2022	<1	51	726	7.02%	<0.001	-2.226	8.02 (3.22-19.97) P<0.001
	1-3	60	1183	5.07%			
	3-6	21	1712	1.23%			
	6-18	5	571	0.88%			
2023	<1	809	1825	44.33%	<0.001	-11.298	3.95 (3.6-4.33) P<0.001
	1-3	727	2288	31.77%			
	3-6	662	3659	18.09%			
	6-18	580	5166	11.23%			
2024	<1	1107	7001	15.81%	<0.001	-4.6	5.83 (5.18-6.58) P<0.001
	1-3	839	8256	10.16%			
	3-6	431	12469	3.46%			
	6-18	327	12067	2.71%			
2019-2024	<1	3552	15457	22.98%	<0.001	-8.348	4.91 (4.59-5.25) P<0.001
	1-3	2918	19358	15.07%			
	3-6	1896	24836	7.63%			
	6-18	995	21269	4.68%

1. Pre-pandemic (2019): Infants (<1 year) had a 46.52-fold higher positivity rate than school-aged children (6–18 years) (26.05% vs. 0.56%).
2. During the pandemic years (2020–2022), the fold difference narrowed: 9.82-fold in 2020 (28.47% vs. 2.90%), 6.66-fold in 2021 (26.82% vs. 4.03%), and 7.98-fold in 2022 (7.02% vs. 0.88%)—underscoring a distinct age-related vulnerability.
3. The post-pandemic rebound in 2023 showed the smallest disparity: infants had a 44.33% positivity rate, 3.95 times that of 6–18-year-olds (11.23%). Even in 2024, despite an overall decline in positivity, infants maintained a 15.81% positivity rate—5.83 times that of the 6–18-year group (2.71%).

To further characterize vulnerability in infants under one year of age, two additional stratified analyses were conducted:

1. A two-group stratification (0–0.5 vs. 0.5–1 year): Significant differences in positivity rates were observed across all years except 2022 (all P<0.001; for 2022: OR = 1.14, 95% CI: 0.64–2.02, P = 0.759, **Figure 1B**, **Supplementary Table S1**).
2. A four-group stratification (0–0.25, 0.25–0.5, 0.5–0.75, 0.75–1 year): Cochran–Armitage tests indicated a significant decreasing trend in positivity rates with increasing age in all years except 2022 (all P<0.001; for 2022: Z = 3.40, P<0.001, slope=0.88). However, the slope of the decline in positivity based on this four-group stratification was less pronounced compared to the original four-age-group analysis (**Table 2** and **Supplementary Table S2**, **Figure 1C**).

Based on these findings, the age-related trend in positivity among infants under one year of age was also disrupted during the pandemic year of 2022.

### NPIs and HRSV positivity dynamics: pandemic-era suppression, post-restriction rebound, and subsequent fluctuations (2019-2024)

**Figure 2:**
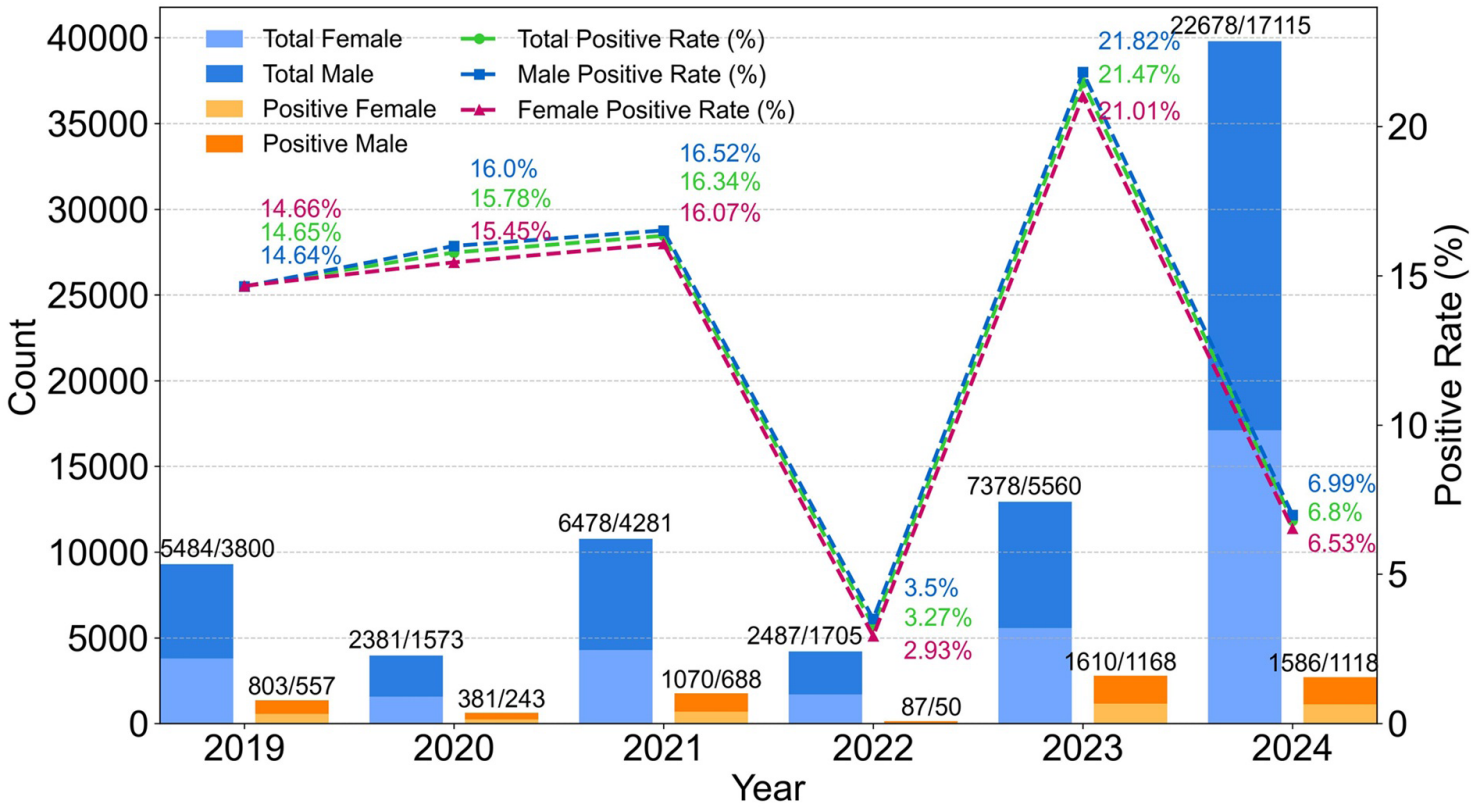
HRSV positivity 2019-2024: overall trends and sex differences. The numbers on the bar chart represent the counts of males and females (males/females), respectively.

There was no statistically significant gender difference in HRSV positivity rate between 2019 and 2024. Notably, only in 2019 did females exhibit a slightly higher positivity rate (14.66%) than males (14.64%), whereas males consistently showed marginally higher rates in all other years (**Table 1**, **Figure 2**).

Annual data revealed that in the pre-pandemic year 2019 and the first two years of the pandemic (2020-2021), the overall positivity rate showed a gradual increase from 14.65% in 2019 to 16.34% in 2021 with minimal fluctuations. However, in 2022—the third year of the pandemic—the positivity rate dropped sharply to 3.27%, which was significantly lower than in any other year (P < 0.001). In 2023, the first year after the lifting of China’s COVID-19 restrictions, there was a rebound surge to a peak of 21.47%, which was significantly higher than in any other year (P < 0.001). Following the year of high positivity, the rate dropped sharply again to 6.80% in 2024, which was significantly lower than the average positivity rate of 15.60% recorded in 2019–2021 (P < 0.001).

### NPIs disrupted seasonal dynamics of HRSV monthly positivity rates in Henan: lockdown suppression, post-restriction shifts, and altered winter peaks

**Figure 3:**
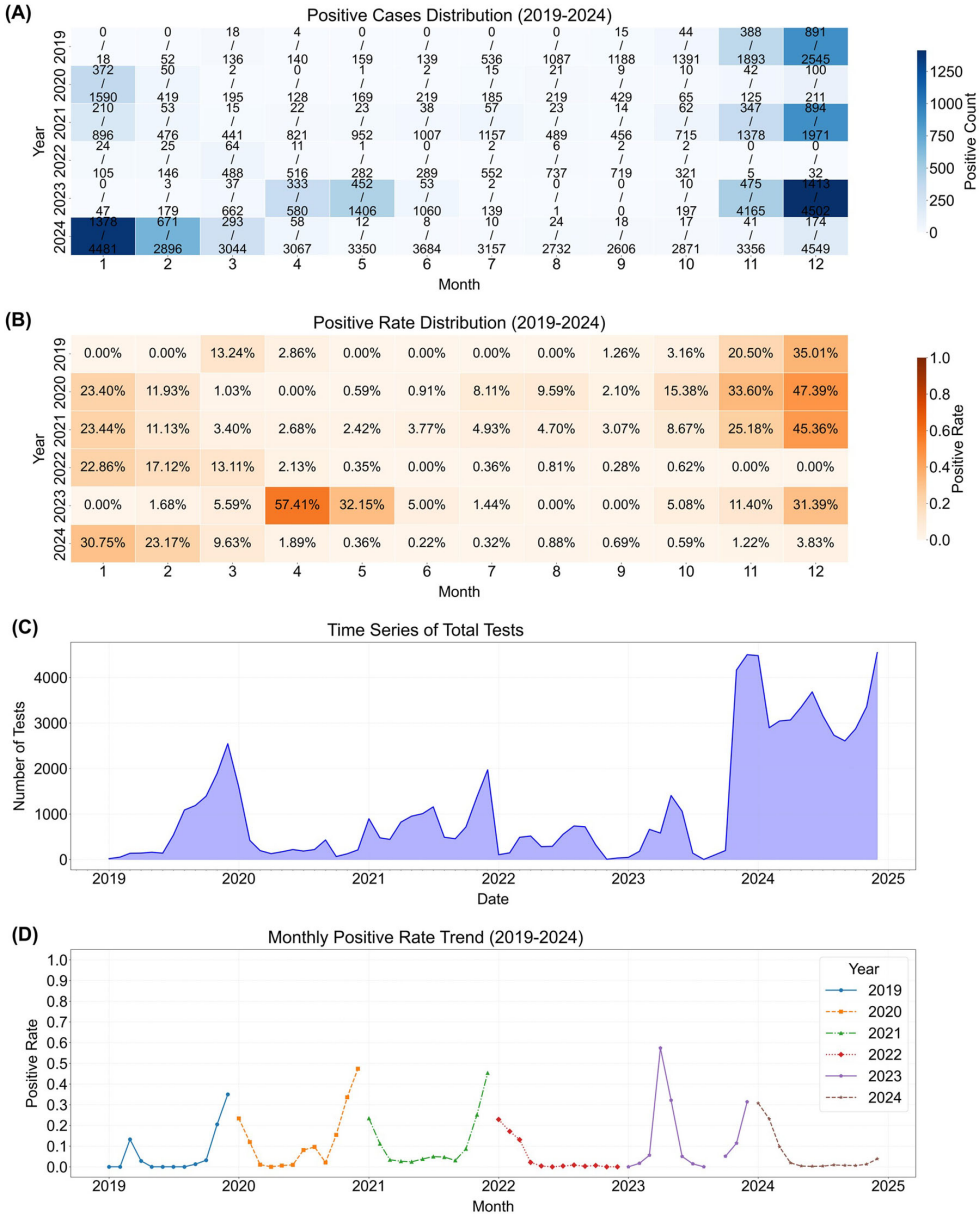
Monthly number of HRSV-positive cases and positivity rate trends. **(A)** Heatmap of monthly HRSV positive cases (top) and total tests (bottom) by year (2019-2024). **(B)** Heatmap of monthly HRSV positive rates (2019-2024) with Oranges colormap. **(C)** Time series of total monthly HRSV tests from 2019 to 2024. **(D)** Monthly HRSV positive rate trends (2019-2024) with distinct line styles for each year.

Monthly data revealed that from 2019 to 2021, positivity rate peaks primarily occurred in winter in Henan, concentrated from November to January of the following year (**Figure 3**). Positive rates increased gradually starting from October each year, persisting until February (and extending to March in 2019 and 2022). Positivity rates remained below 5% in most other months, except for July 2020 (8.11%) and August 2020 (9.59%).

However, during strict lockdowns in central China in 2022, the previously observed high positivity rates were absent from October 2022 to February 2023. Notably, following the lifting of restrictions in 2023, extremely high positivity rates emerged in April (57.41%) and May (32.15%). Subsequently, the high-prevalence season occurred again from October 2023 to February 2024, but with rates significantly lower compared to 2019–2021—particularly in November (11.40% vs. 25.18% in 2021, P < 0.001) and December (31.39% vs. 45.36% in 2021, P < 0.001).

In 2024, no high positivity rates were observed from October to December, with rates remaining only between 0.59% and 3.83%, which were also significantly lower than the corresponding periods in 2019–2021 (P < 0.001).

### Quantitative impact of policy interventions: interrupted time-series analysis

To quantitatively evaluate the impact of COVID-19 policy interventions on HRSV epidemiology, we conducted an interrupted time-series analysis using a negative binomial generalized linear model. The model demonstrated excellent fit (pseudo R²=0.446, AIC = 661.99; **Supplementary Table S3**) and revealed significant disruptions at both intervention points (**Supplementary Table S4**).

**Figure 4:**
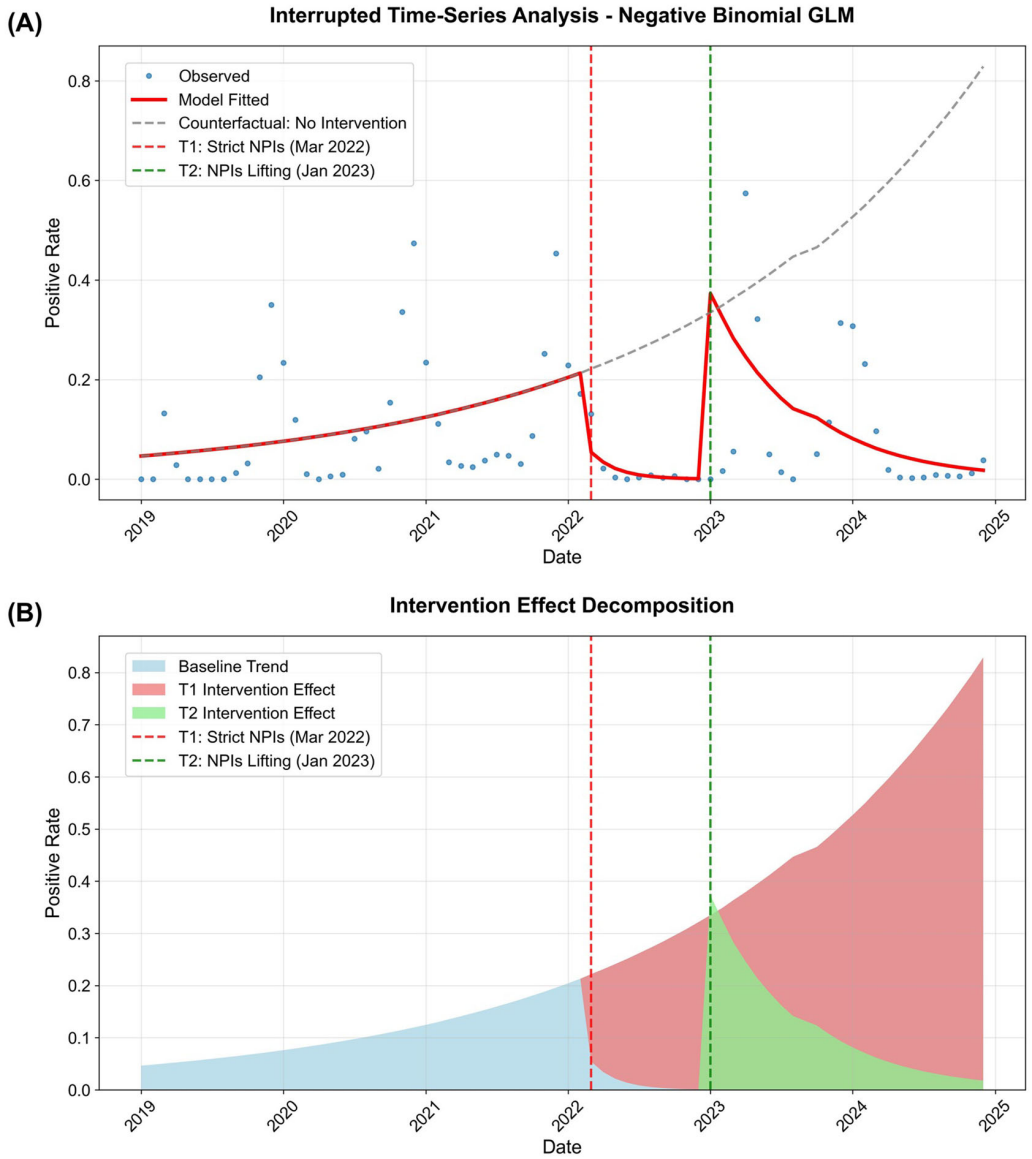
Interrupted time-series analysis of HRSV positivity rates and intervention effect decomposition. **(A)** Main results of interrupted time-series analysis using negative binomial generalized linear model with robust standard errors (HC3). Blue circles represent observed HRSV positivity rates, red solid line shows model-fitted values, and gray dashed line indicates the counterfactual scenario assuming no interventions. Vertical dashed lines mark the two key intervention points: T1 (March 2022, representing strict NPIs implementation) and T2 (January 2023, representing NPIs lifting). **(B)** Decomposition of intervention effects on HRSV positivity rates. The stacked areas represent the contributions of baseline trend (light blue), T1 intervention effect (light coral), and T2 intervention effect (light green). Vertical dashed lines indicate the intervention points as described in panel A.

The implementation of strict NPIs in March 2022 (T1) was associated with a significant negative trend change in HRSV positivity (coefficient: -0.498, P = 0.005), indicating a progressive decline in viral circulation following intervention implementation. Although the immediate level change at T1 did not reach statistical significance (coefficient: -1.415, P = 0.064), the overall trend reversal was highly significant. Conversely, the lifting of NPIs in January 2023 (T2) produced an immediate and substantial surge in positivity rates (coefficient: 6.505, P<0.001), representing a 668.77-fold increase in infection odds (95% CI: 47.03-9509.28) compared to the counterfactual scenario (**Figure 4A**, **Supplementary Table S4**).

Counterfactual analysis provided compelling evidence of intervention effectiveness (**Supplementary Table S5**). At 6 months post-T1, the actual positivity rate was 0.55% compared to the counterfactual prediction of 27.27%, representing a 97.99% relative reduction (absolute effect: -26.72%). The temporal evolution of these effects is visualized in **Figure 4B**, which demonstrates the progressive impact of both interventions over time. Following T2, the initial surge (37.27% actual vs. 33.50% counterfactual at 1 month post-T2; +11.25% relative effect) was followed by a gradual decline, with a 84.54% relative reduction observed at 12 months post-intervention compared to counterfactual predictions.

The model’s robustness was confirmed through comprehensive diagnostic analyses. Residual diagnostics (**Supplementary Figure S1**) showed no systematic patterns, and the Q-Q plot (**Supplementary Figure S2**) indicated normally distributed residuals. The coefficient forest plot (**Supplementary Figure S3**) visually demonstrates the magnitude and precision of each parameter estimate, while the odds ratio forest plot (**Supplementary Figure S4**) highlights the substantial impact of T2 intervention lifting.

Supporting analyses further contextualized these findings. The total testing volume remained substantial throughout the study period (**Supplementary Figure S5**), indicating that observed patterns reflect true epidemiological changes rather than surveillance artifacts. The moving average trends (**Supplementary Figure S6**) corroborated the ITSA findings, showing clear suppression during T1 and resurgence following T2. Seasonal analysis (**Supplementary Figure S7**) revealed altered periodicity, with the characteristic winter peak disrupted and replaced by atypical spring outbreaks during the transition period.

The relationship between testing volume and positivity rates (**Supplementary Figure S8**) showed distinct clustering by intervention period, with no evidence of positive correlation between testing intensity and detection rates. The historic low positivity rate occurred in 2022 (3.27%) under strict NPIs, despite modest testing volume. Conversely, the highest testing volume in 2024 (n=39,793) coincided with a significantly reduced positivity rate (6.80%) compared to the 2019–2021 average (15.60%; P<0.001) (**Supplementary Figure S5**, **S9**). This dissociation confirms that observed patterns reflect true epidemiological changes rather than surveillance artifacts. The counterfactual comparison (**Supplementary Figure S10**) illustrates how intervention timing critically shaped the epidemic trajectory.

### Quantitative impact of policy interventions on seasonality and transmission dynamics

#### Seasonal decomposition reveals disrupted epidemic patterns

**Figure 5:**
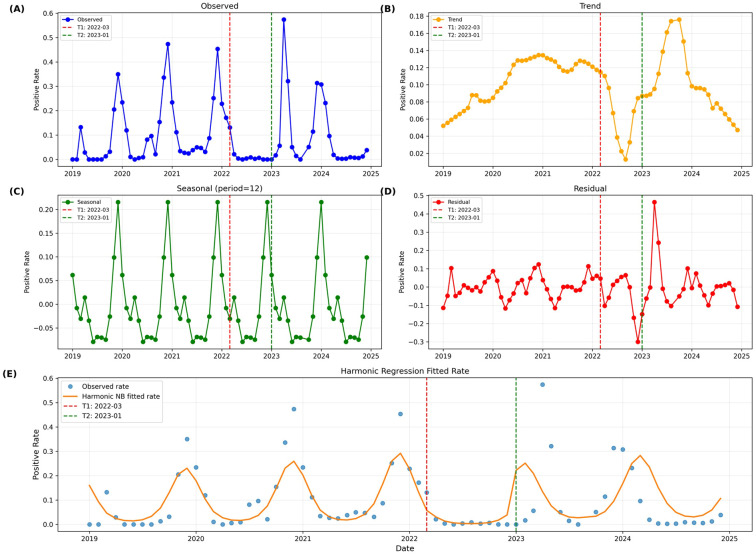
Seasonal decomposition and harmonic regression analysis of HRSV positivity rates, 2019-2024. **(A)** Observed positivity rates with policy intervention timepoints (T1: March 2022, strict NPIs; T2: January 2023, NPI lifting). **(B)** Trend component showing suppression-rebound-decline pattern. **(C)** Seasonal component demonstrating disrupted winter patterns and anomalous spring peaks. **(D)** Residual component identifying significant deviations during 2023 resurgence. **(E)** Harmonic regression fitted rates quantifying seasonal amplitude and phase shifts.

Classical additive seasonal decomposition of HRSV positivity rates (2019-2024) quantified profound disruptions in epidemic dynamics (**Figures 5A–D**). The trend component demonstrated the characteristic “suppression-rebound-decline” pattern: gradual increase during 2019-2021 (peak ~0.15), sharp suppression during 2022 NPIs (trough ~0.01), rapid rebound post-NPI lifting in 2023 (peak ~0.35), and subsequent decline to ~0.05 in 2024, closely aligning with immune debt theory.

Seasonal components revealed distinct patterns across intervention periods (**Supplementary Table S6**). During 2019-2021, stable winter epidemic patterns prevailed with November-January peaks (maximum: 0.215 in December 2019). These patterns disappeared during strict NPIs in 2022, when seasonal fluctuations diminished dramatically (range: ± 0.03 vs ±0.15 baseline). Post-NPI lifting, seasonal patterns re-emerged but with altered characteristics, including anomalous spring peaks in April-May 2023. Residual analysis identified significant positive deviations during the 2023 resurgence (April: 0.464, May: 0.243, **Supplementary Table S7**), confirming positivity rates substantially exceeded seasonal expectations.

#### Harmonic regression quantifies seasonal amplitude and phase shifts

Negative binomial harmonic regression (pseudo R²=0.6057) systematically quantified seasonal characteristic changes (**Figure 5E**, **Supplementary Table S8**). Seasonal amplitude varied significantly across intervention periods (χ²=8.74, P = 0.013), increasing from baseline (1.371, 95% CI: 1.082-1.660) during strict NPIs (1.637, 95% CI: 1.215-2.059) then declining post-NPI lifting (1.252, 95% CI: 0.987-1.517). Dramatic phase shifts occurred (F = 12.39, P<0.001): the epidemic peak advanced from mid-January (1.213 months, baseline) to early November (11.098 months) during strict NPIs, then partially recovered to late January/early February (1.912 months) post-NPI lifting.

The complete harmonic regression coefficient table (**Supplementary Table S9**) demonstrated that strict NPIs (post_T1: -1.669, P = 0.003; sin1_T1: 1.558, P = 0.006) significantly affected both positivity levels and seasonal phase, while NPI lifting effects reflected natural restoration rather than additional intervention-driven shifts.

#### Count regression models confirm intervention effects on transmission dynamics

Comparative analysis of negative binomial and quasi-Poisson models (**Supplementary Table S10**) favored the negative binomial approach for analyzing monthly HRSV-positive counts (offset by log tests). The negative binomial model provided robust estimates with superior fit (pseudo R²=0.7136) compared to the overdispersed quasi-Poisson model (dispersion=46.077).

The negative binomial model revealed a significant baseline time trend (coefficient: 0.0358, P = 0.025), equivalent to a 3.6% monthly increase absent interventions. Policy effects were substantial: strict NPIs produced a progressive 53.2% monthly decrease in positive counts (time_after_T1: -0.5315, P = 0.002), while NPI lifting caused an immediate 998.8% surge (post_T2: 6.9976, P<0.001) with continuing upward trend (time_after_T2: 0.2884, P = 0.100), strongly supporting immune debt dynamics. Significant seasonal terms (cos1: 1.1150, P<0.001; sin1: -0.6512, P<0.001) confirmed stable annual fluctuation patterns.

**Figure 6:**
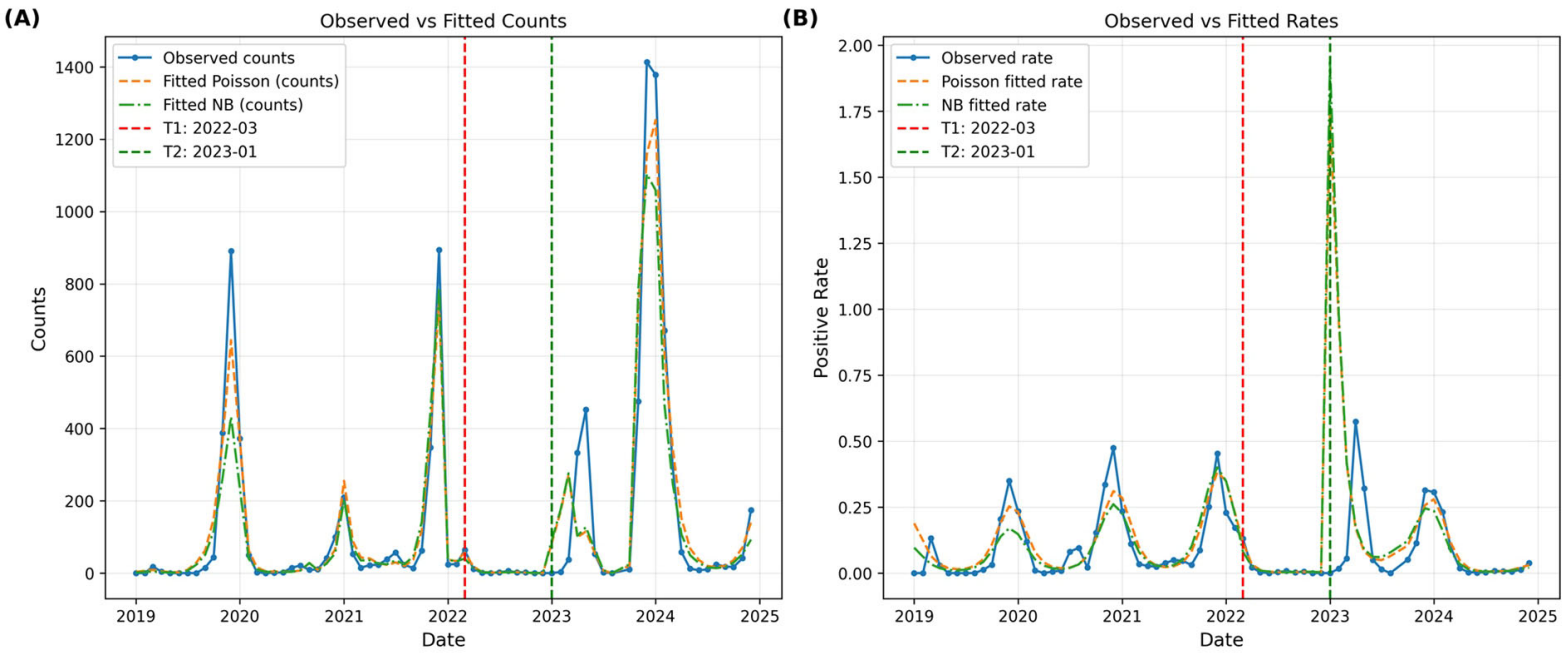
Count regression model fits for HRSV-positive cases and rates, 2019-2024. **(A)** Observed versus fitted positive counts using negative binomial and quasi-Poisson models. **(B)** Observed versus fitted positivity rates, demonstrating accurate capture of epidemic patterns across intervention periods.

The complete coefficient comparison (**Supplementary Table S11**) demonstrated consistent directionality between models, with the negative binomial model providing more precise estimates and superior statistical significance across key parameters. Conversion to positivity rates demonstrated excellent model fit across all periods (**Figures 6A, B**), accurately capturing the 2019–2021 fluctuations, 2022 suppression, 2023 spring outbreak (April fitted: 0.57 vs observed: 57.41%), and 2024 decline.

#### Multivariate logistic regression analysis

**Figure 7:**
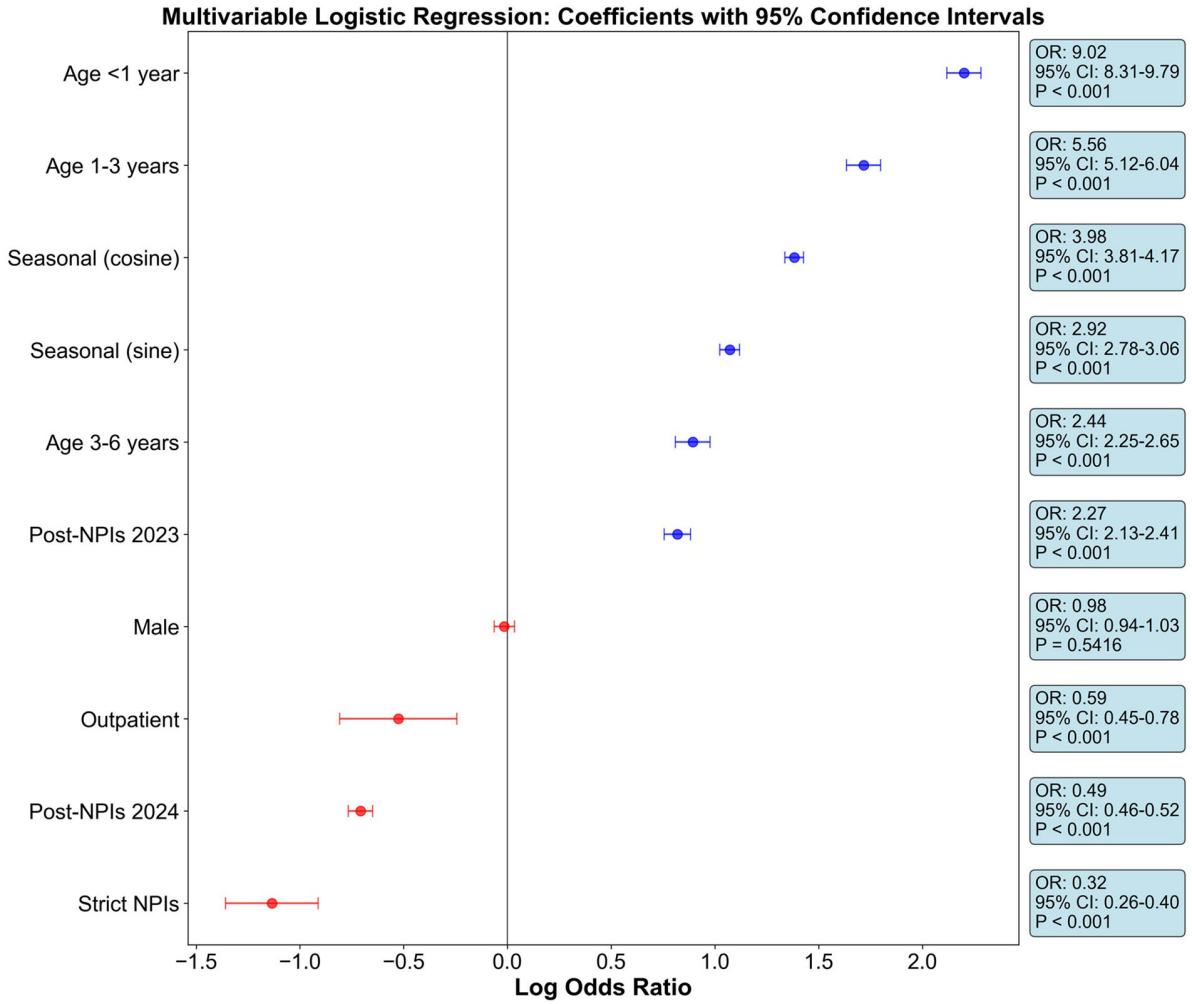
Forest plot of multivariate logistic regression analysis for HRSV infection risk in children. This figure shows the adjusted odds ratios (OR) and their 95% confidence intervals (CI) for each variable in the multivariate logistic regression model. All age groups were referenced to school-age children (6–18 years). The policy intervention variable was referenced to the pre-intervention period (2019–February 2022), including the strict NPIs period (March–December 2022), the first year after NPIs lifting (2023), and the second year after NPIs lifting (2024). Patient type was referenced to inpatients. All P values remained statistically significant (P<0.001) after FDR correction.

To comprehensively evaluate the independent effects of age, gender, seasonality, and policy interventions on the risk of HRSV infection, we constructed a multivariate logistic regression model (**Supplementary Table S12**, **Figure 7**). The model exhibited good fit (pseudo-R²=0.227, AIC = 44836.02) and could explain 22.7% of the variation in HRSV infection risk.

Age was the strongest independent predictor of HRSV infection. Compared with school-age children (6–18 years), infants (<1 year) had the highest infection risk (OR = 9.02, 95%CI: 8.31–9.79, P<0.001), indicating that infants were 9 times more likely to be infected with HRSV than school-age children. Children aged 1–3 years had the second-highest infection risk (OR = 5.56, 95%CI: 5.12–6.04, P<0.001), while those aged 3–6 years also showed a significantly higher infection risk than school-age children (OR = 2.44, 95%CI: 2.25–2.65, P<0.001). This age gradient highlights the extreme vulnerability of young children, especially infants under 1 year old, to HRSV infection.

Policy interventions showed a significant independent effect. During the period of strict NPIs implementation (March–December 2022), the risk of HRSV infection was significantly reduced (OR = 0.32, 95%CI: 0.26–0.40, P<0.001), with the infection risk being only 32% of that before the intervention. In contrast, after the lifting of NPIs in 2023, the infection risk increased significantly (OR = 2.27, 95%CI: 2.13–2.41, P<0.001), which was 2.27 times the risk before the intervention. Notably, the infection risk in 2024 fell back to 49% of the pre-intervention level (OR = 0.49, 95%CI: 0.46–0.52, P<0.001), suggesting that the epidemic pattern is gradually returning to normal.

Seasonality also showed statistical significance. Both the sine term (OR = 2.92, 95%CI: 2.78–3.06, P<0.001) and cosine term (OR = 3.98, 95%CI: 3.81–4.17, P<0.001) confirmed the strong seasonal fluctuation pattern of HRSV infection. After adjusting for other variables, gender had no significant statistical significance (OR = 0.99, 95%CI: 0.94–1.03, P = 0.542), while outpatients had a significantly lower infection risk than inpatients (OR = 0.59, 95%CI: 0.45–0.78, P<0.001).

## Discussion

This six-year surveillance study from Central China provides compelling and multi-faceted evidence of the profound disruption to human respiratory syncytial virus (HRSV) epidemiology caused by non-pharmaceutical interventions (NPIs) during the COVID-19 pandemic. By integrating classical epidemiological analyses with advanced quantitative methods—including interrupted time-series analysis, seasonal decomposition, harmonic regression, and multivariate modeling—we have precisely quantified the magnitude, timing, and dynamics of these disruptions. Our findings robustly document a period of intense viral suppression followed by an unprecedented, off-season resurgence in 2023, a pattern that strongly aligns with and provides quantitative support for the “Immune debt” hypothesis. This theory posits that reduced pathogen exposure, particularly among immunologically naive young children, leads to an accumulation of susceptible individuals, thereby fueling post-intervention epidemics of greater intensity and altered timing (Hatter et al., 2021; Cohen et al., 2023; Munro and House, 2024).

### NPI-driven suppression and post-lifting resurgence: quantitative validation

Before 2022, HRSV positivity rates remained stable (14.65% in 2019, 16.34% in 2021), but strict NPIs in 2022 pushed rates to a historic low of 3.27%—only a quarter of pre-pandemic levels. This starkly illustrates the effectiveness of measures like masking and social distancing in curbing the transmission of respiratory viruses (Ravkin et al., 2022; Lenglart et al., 2023; Leija-Martinez et al., 2024). This suppression was followed by a dramatic rebound to 21.47% in 2023, post-NPI relaxation. This increase, representing a 6.5-fold rise compared to 2022, underscores the accumulation of “Immune debt” in the population—particularly among young children, who were unable to develop natural immunity due to NPIs (Munro and House, 2024; Rubin, 2024).

Our ITSA, using a negative binomial generalized linear model with excellent fit (pseudo R²=0.446), confirmed these trends causally (**Figure 4**; **Supplementary Table S3**). Implementation of strict NPIs in March 2022 (T1) was associated with a significant downward trend in positivity (coefficient: -0.498, P = 0.005), reflecting progressive reduction in viral circulation. Conversely, NPI lifting in January 2023 (T2) triggered an immediate 668.77-fold increase in infection odds (95% CI: 47.03–9509.28, P<0.001). Counterfactual analysis further underscored NPI effectiveness: 6 months post-T1, the actual positivity rate (0.55%) was 97.99% lower than the predicted rate (27.27%) in the absence of interventions (**Supplementary Table S5**).

Complementary count regression models (negative binomial, pseudo R²=0.7136) accurately captured these dynamics, including the anomalous April 2023 spring peak (fitted rate: 0.57 vs. observed: 57.41%) and the 2024 decline (**Figure 6**; **Supplementary Table S10**). The model revealed strict NPIs reduced monthly positive counts by 53.2% (P = 0.002), while NPI lifting caused an immediate 998.8% surge (P<0.001)—findings aligned with global observations of post-NPI HRSV resurgences and validating the role of accumulated immune debt (Foley et al., 2021; Garg et al., 2022; Quintero-Salgado et al., 2024).

The significant rebound of HRSV infections in 2023 is consistent with the immune debt hypothesis, which is further supported by modeling analyses in this study (ITSA, pseudo R² = 0.446). However, other factors may have also contributed to the observed resurgence. First, changes in testing practices and healthcare utilization may have played a role. Our data show that while testing volumes in 2019, 2021, and 2023 were 9,284, 10,759, and 12,938, respectively—only a modest increase in 2023 compared to previous years—the positivity rate rose significantly from 14.65% and 16.34% to 21.47% (χ² = 198.36, df = 2, P < 0.001). In 2024, testing volume surged to 39,793—a 207.6% increase over 2023—yet the positivity rate dropped to 6.80%, significantly lower than the 2019–2021 average of 15.60% and the 2023 rate of 21.47% (χ² = 2146.52, df = 2, P < 0.001). This indicates no direct correlation between testing intensity and positivity rates in this study. Second, trends in other respiratory pathogens during the same period provide context. Influenza data from the same hospital revealed 40,785 cases in 2023—a 252.9% increase over 2022—while regional surveillance showed a Mycoplasma pneumoniae positivity rate of 35.01% in 2023, suggesting a broader resurgence of multiple pathogens (Jia et al., 2024; Qi et al., 2025). Nevertheless, the intensity of the HRSV rebound (21.47%, 6.57 times that of 2022) and its atypical timing (peaking in April at 57.41%, earlier than the autumn–winter peaks of influenza and M. pneumoniae) remain highly consistent with the immune debt mechanism, wherein accumulated susceptibility among infants and young children during NPIs led to concentrated infection after restrictions were lifted. Third, behavioral changes such as increased social gathering after the relaxation of NPIs may have also facilitated transmission.

### Disrupted seasonality: quantified shifts in amplitude and phase

HRSV’s typical winter peak (November–January) disappeared in 2022, replaced by an extreme spring outbreak in 2023 (April positivity: 57.41%). This temporal shift is likely a direct consequence of altered population immunity and transmission dynamics following the widespread lifting of NPIs (Sun et al., 2024; Heemskerk et al., 2025). Seasonal decomposition (**Figures 5A–D**) clarified this disruption: the trend component showed a “suppression-rebound-decline” pattern (2019–2021 peak: ~0.15; 2022 trough: ~0.01; 2023 rebound: ~0.35; 2024 decline: ~0.05). Seasonal fluctuations shrank from ±0.15 (2019–2021 baseline) to ±0.03 during 2022 NPIs, with significant positive residuals during the 2023 spring peak (April: 0.464, May: 0.243; **Supplementary Table S7**), confirming these outbreaks exceeded seasonal expectations.

Harmonic regression (pseudo R²=0.6057) quantified key shifts (**Figure 5E**; **Supplementary Table S8**). Seasonal amplitude varied significantly across periods (χ²=8.74, P = 0.013): increasing from 1.371 (baseline, 95% CI: 1.082–1.660) to 1.637 (strict NPIs, 95% CI: 1.215–2.059) before declining to 1.252 post-NPIs (95% CI: 0.987–1.517). The epidemic peak also shifted dramatically (F = 12.39, P<0.001): from mid-January (1.213 months, baseline) to early November (11.098 months) during NPIs, then partially recovering to late January/early February (1.912 months) post-lifting.

### Age-specific susceptibility: multivariate confirmation

Infants <1 year remained the highest-risk group (overall positivity: 22.98%), with a 4.91-fold higher rate than school-aged children (6–18 years: 4.68%; P<0.001; **Table 2**; **Figure 1A**). In 2023, their positivity reached 44.33% (3.95 times that of 6–18-year-olds), and even in 2024, it remained 15.81% (5.83 times higher).

Multivariate logistic regression (**Figure 7**; **Supplementary Table S11**) identified age as the strongest predictor: compared to 6–18-year-olds, infants <1 year had an odds ratio (OR) of 9.02 (95% CI: 8.31–9.79, P<0.001), followed by 1–3-year-olds (OR = 5.56, 95% CI: 5.12–6.04, P<0.001) and 3–6-year-olds (OR = 2.44, 95% CI: 2.25–2.65, P<0.001). Notably, 2022 NPIs disrupted the typical “younger = higher risk” gradient in infants: no difference between 0–0.5 and 0.5–1-year-olds (OR = 1.14, P = 0.759), and a reversed slope in 4-subgroup analysis (slope=0.88, P<0.001; **Figures 1B, C**). This flattening reflected extreme viral suppression, creating a uniformly susceptible infant cohort that fueled the 2023 resurgence.

### The 2024 decline: partial repayment of immune debt

HRSV positivity fell to 6.80% in 2024—significantly below the 2019–2021 average (15.60%, P<0.001; **Table 1**; **Figure 2**). This decline likely reflects partial “repayment” of immune debt: widespread 2023 infections (21.47% positivity) rebuilt population immunity, particularly in previously naive infants, reducing the susceptible pool. While viral competition (e.g., Mycoplasma pneumoniae) or residual behavioral changes may contribute, the timing and magnitude of the decline align with immune reconstitution expectations. Long-term surveillance is needed to confirm if this signals a return to pre-pandemic patterns.

### Implications for public health

Our findings underscore the urgent need for public-health systems to recalibrate for the post-pandemic era. The 2023 spring surge demonstrates that season-centric preparedness is no longer adequate (Quintero-Salgado et al., 2024); instead, adaptive, data-driven strategies are essential. We recommend: (1) embedding quantitative tools like interrupted time-series analysis into routine forecasting; (2) maintaining year-round, multi-pathogen surveillance to detect off-season spikes; (3) prioritizing infant protection through the deployment of novel tools such as nirsevimab and maternal HRSV vaccination, given their about five-fold higher infection risk; and (4) integrating HRSV monitoring with influenza and Mycoplasma pneumoniae surveillance. These measures will strengthen resilience against co-circulating respiratory threats.

### Limitations of the study

This study has several limitations. First, its single-center design, based on data from Henan Children’s Hospital, may limit the generalizability of the findings to the broader Central China region, despite the hospital’s substantial coverage of the pediatric population in the area. Selection bias may also be present, as the study included only children seeking care at a tertiary hospital, which may not represent the broader community or those with milder or asymptomatic infections. In addition, changes in testing protocols and public health policies over the study period (2019–2024) could have influenced HRSV detection rates and temporal trends. Furthermore, the lack of data on disease severity and clinical outcomes restricts deeper insight into the clinical burden and progression of HRSV infections. Future multi-center studies incorporating diverse healthcare settings and detailed clinical data are needed to validate and extend these findings.

## Data Availability

The original contributions presented in the study are included in the article/**Supplementary Material**. Further inquiries can be directed to the corresponding author.
