# Supplementary material for: Six-year epidemiological dynamics of human respiratory syncytial virus infections in children in central China (2019-2024): pandemic suppression, 2023 resurgence, and immune debt effect
Source: Front Cell Infect Microbiol. 2026 Jan 13;15:1691957. doi: 10.3389/fcimb.2025.1691957 (PMC12835292; doi:10.3389/fcimb.2025.1691957)
Supplement: Supplementary file 1 [file DataSheet1.docx]

Table S1 Statistical analysis results of HRSV positivity rates in infants aged <1 year (stratified into 0-0.5 years vs. 0.5-1 year)

| Year | Chi2 | P_chi2 | OR | 95%CI |
| --- | --- | --- | --- | --- |
| 2019 | 10.932 | <0.001 | 1.357 | 1.135_1.622 |
| 2020 | 12.267 | <0.001 | 1.561 | 1.221_1.995 |
| 2021 | 29.367 | <0.001 | 1.632 | 1.368_1.947 |
| 2022 | 0.094 | 0.759 | 1.142 | 0.644_2.025 |
| 2023 | 26.379 | <0.001 | 1.607 | 1.343_1.924 |
| 2024 | 42.517 | <0.001 | 1.541 | 1.353_1.754 |

Table S2 Statistical analysis results of HRSV positivity rates in infants aged <1 year (stratified into four subgroups: 0-0.25, 0.25-0.5, 0.5-0.75, and 0.75-1 year)

| Year | Cochran_Armitage_Z | Cochran_Armitage_P | Slope | R_squared |
| --- | --- | --- | --- | --- |
| 2019 | -236.826 | <0.001 | -3.849 | 0.865 |
| 2020 | -121.094 | <0.001 | -3.812 | 0.884 |
| 2021 | -302.193 | <0.001 | -4.204 | 0.679 |
| 2022 | 3.396 | <0.001 | 0.882 | 0.165 |
| 2023 | -218.987 | <0.001 | -4.889 | 0.869 |
| 2024 | -661.044 | <0.001 | -2.721 | 0.951 |

Table S3 Interrupted Time-Series Analysis Model Goodness-of-Fit Statistics

| Statistic | Value |
| --- | --- |
| Model Type | Negative Binomial Generalized Linear Model |
| Pseudo R² | 0.446 |
| AIC | 661.99 |
| BIC | -159.17 |
| Log Likelihood | -324.99 |
| Number of Observations | 71 |
| Number of Parameters | 6 |

Table S4 Coefficients and Effect Estimates from the Interrupted Time-Series Regression Model

| Variable | Coefficient | Standard Error | P-value | OR | 95% CI |
| --- | --- | --- | --- | --- | --- |
| Intercept | -3.07 | 0.341 | <0.001 | 0.046 | (0.024, 0.091) |
| Time Trend | 0.041 | 0.016 | 0.008 | 1.042 | (1.011, 1.074) |
| T1 Intervention Immediate Effect | -1.415 | 0.763 | 0.064 | 0.243 | (0.054, 1.084) |
| T1 Intervention Trend Effect | -0.498 | 0.179 | 0.005 | 0.608 | (0.428, 0.863) |
| T2 Intervention Immediate Effect | 6.505 | 1.354 | <0.001 | 668.772 | (47.034, 9509.278) |
| T2 Intervention Trend Effect | 0.319 | 0.181 | 0.078 | 1.376 | (0.965, 1.962) |

Table S5 Quantitative analysis of the effects of NPI interventions on HRSV positivity rates

| Intervention | Months After Intervention | Actual Positive  Rate (%) | Counterfactual Prediction (%) | Absolute Effect (%) | Relative Effect (%) |
| --- | --- | --- | --- | --- | --- |
| T1 | 1 | 5.39 | 22.19 | -16.8 | -75.7 |
| T1 | 3 | 2.16 | 24.1 | -21.94 | -91.03 |
| T1 | 6 | 0.55 | 27.27 | -26.72 | -97.99 |
| T1 | 12 | 32.46 | 34.91 | -2.45 | -7.02 |
| T2 | 1 | 37.27 | 33.5 | 3.77 | 11.25 |
| T2 | 3 | 28.26 | 36.37 | -8.11 | -22.29 |
| T2 | 6 | 18.67 | 41.16 | -22.49 | -54.64 |
| T2 | 12 | 8.14 | 52.69 | -44.54 | -84.54 |

Table S6 Core statistics of HRSV positivity rate seasonal decomposition (2019–2024)

| Period | Time Range | Trend Component (Mean ± SD) | Seasonal Component (Peak/Trough) | Residual Component (95% Reference Range) |
| --- | --- | --- | --- | --- |
| Baseline (Pre-T1) | 2019-01~2022-02 | 0.082 ± 0.031 | 0.215 (2019-12) / -0.079 (2019-06) | ±0.052 |
| Strict NPI Period (T1–T2) | 2022-03~2022-12 | 0.023 ± 0.018 | 0.028 (2022-02) / -0.030 (2022-05) | ±0.037 |
| NPI Lifted Period (Post-T2) | 2023-01~2024-12 | 0.115 ± 0.087 | 0.062 (2023-04) / -0.068 (2024-07) | ±0.124 |

Table S7 HRSV positivity rate seasonal decomposition components (January 2019–December 2024)

| date | observed | trend | seasonal | resid | date | observed | trend | seasonal | resid |
| --- | --- | --- | --- | --- | --- | --- | --- | --- | --- |
| 2019-1 | 0 | 0.052 | 0.062 | -0.114 | 2022-1 | 0.229 | 0.121 | 0.062 | 0.046 |
| 2019-2 | 0 | 0.056 | -0.008 | -0.048 | 2022-2 | 0.171 | 0.117 | -0.008 | 0.062 |
| 2019-3 | 0.132 | 0.059 | -0.03 | 0.104 | 2022-3 | 0.131 | 0.115 | -0.03 | 0.047 |
| 2019-4 | 0.029 | 0.063 | 0.015 | -0.048 | 2022-4 | 0.021 | 0.11 | 0.015 | -0.103 |
| 2019-5 | 0 | 0.066 | -0.034 | -0.032 | 2022-5 | 0.004 | 0.096 | -0.034 | -0.058 |
| 2019-6 | 0 | 0.069 | -0.079 | 0.01 | 2022-6 | 0 | 0.067 | -0.079 | 0.012 |
| 2019-7 | 0 | 0.073 | -0.069 | -0.004 | 2022-7 | 0.004 | 0.039 | -0.069 | 0.034 |
| 2019-8 | 0 | 0.088 | -0.07 | -0.018 | 2022-8 | 0.008 | 0.023 | -0.07 | 0.056 |
| 2019-9 | 0.013 | 0.088 | -0.075 | -0.001 | 2022-9 | 0.003 | 0.013 | -0.075 | 0.064 |
| 2019-10 | 0.032 | 0.081 | -0.026 | -0.024 | 2022-10 | 0.006 | 0.033 | -0.026 | -0.001 |
| 2019-11 | 0.205 | 0.08 | 0.099 | 0.026 | 2022-11 | 0 | 0.069 | 0.099 | -0.168 |
| 2019-12 | 0.35 | 0.081 | 0.215 | 0.054 | 2022-12 | 0 | 0.085 | 0.215 | -0.3 |
| 2020-1 | 0.234 | 0.085 | 0.062 | 0.087 | 2023-1 | 0 | 0.087 | 0.062 | -0.149 |
| 2020-2 | 0.119 | 0.092 | -0.008 | 0.035 | 2023-2 | 0.017 | 0.087 | -0.008 | -0.063 |
| 2020-3 | 0.01 | 0.097 | -0.03 | -0.056 | 2023-3 | 0.056 | 0.089 | -0.03 | -0.003 |
| 2020-4 | 0 | 0.102 | 0.015 | -0.117 | 2023-4 | 0.574 | 0.095 | 0.015 | 0.464 |
| 2020-5 | 0.006 | 0.113 | -0.034 | -0.072 | 2023-5 | 0.321 | 0.113 | -0.034 | 0.243 |
| 2020-6 | 0.009 | 0.123 | -0.079 | -0.035 | 2023-6 | 0.05 | 0.139 | -0.079 | -0.01 |
| 2020-7 | 0.081 | 0.128 | -0.069 | 0.022 | 2023-7 | 0.014 | 0.161 | -0.069 | -0.078 |
| 2020-8 | 0.096 | 0.128 | -0.07 | 0.038 | 2023-8 | 0 | 0.174 | -0.07 | -0.104 |
| 2020-9 | 0.021 | 0.129 | -0.075 | -0.033 | 2023-10 | 0.051 | 0.176 | -0.075 | -0.051 |
| 2020-10 | 0.154 | 0.131 | -0.026 | 0.049 | 2023-11 | 0.114 | 0.151 | -0.026 | -0.011 |
| 2020-11 | 0.336 | 0.133 | 0.099 | 0.104 | 2023-12 | 0.314 | 0.114 | 0.099 | 0.101 |
| 2020-12 | 0.474 | 0.135 | 0.215 | 0.124 | 2024-1 | 0.308 | 0.098 | 0.215 | -0.006 |
| 2021-1 | 0.234 | 0.135 | 0.062 | 0.038 | 2024-2 | 0.232 | 0.096 | 0.062 | 0.074 |
| 2021-2 | 0.111 | 0.131 | -0.008 | -0.012 | 2024-3 | 0.096 | 0.096 | -0.008 | 0.008 |
| 2021-3 | 0.034 | 0.13 | -0.03 | -0.065 | 2024-4 | 0.019 | 0.095 | -0.03 | -0.045 |
| 2021-4 | 0.027 | 0.127 | 0.015 | -0.115 | 2024-5 | 0.004 | 0.088 | 0.015 | -0.099 |
| 2021-5 | 0.024 | 0.121 | -0.034 | -0.062 | 2024-6 | 0.002 | 0.073 | -0.034 | -0.036 |
| 2021-6 | 0.038 | 0.116 | -0.079 | 0 | 2024-7 | 0.003 | 0.078 | -0.079 | 0.004 |
| 2021-7 | 0.049 | 0.115 | -0.069 | 0.003 | 2024-8 | 0.009 | 0.072 | -0.069 | 0.006 |
| 2021-8 | 0.047 | 0.118 | -0.07 | -0.001 | 2024-9 | 0.007 | 0.066 | -0.07 | 0.011 |
| 2021-9 | 0.031 | 0.124 | -0.075 | -0.019 | 2024-10 | 0.006 | 0.06 | -0.075 | 0.021 |
| 2021-10 | 0.087 | 0.128 | -0.026 | -0.016 | 2024-11 | 0.012 | 0.053 | -0.026 | -0.016 |
| 2021-11 | 0.252 | 0.127 | 0.099 | 0.026 | 2024-12 | 0.038 | 0.047 | 0.099 | -0.108 |
| 2021-12 | 0.454 | 0.124 | 0.215 | 0.114 |  |  |  |  |  |

Table S8 Seasonal amplitude, phase, and key policy coefficients of the harmonic regression model

| Indicator | Baseline (Pre-T1) | Strict NPI Period (Post-T1) | NPI Lifted Period (Post-T2) | Statistical Significance (P-value) |
| --- | --- | --- | --- | --- |
| Seasonal Amplitude (95% CI) | 1.371  (1.082–1.660) | 1.637  (1.215–2.059) | 1.252  (0.987–1.517) | Amplitude difference: χ²=8.74, P=0.013 |
| Seasonal Phase (Month, 95% CI) | 1.213  (0.985–1.441) | 11.098  (10.762–11.434) | 1.912  (1.653–2.171) | Phase shift: F=12.39, P<0.001 |
| Core Policy Coefficients (Negative Binomial Model) | — | post_T1: -1.669  (-2.778–-0.561) | post_T2: 1.603 (0.555–2.650) | post_T1: P=0.003; post_T2: P=0.003 |

Table S9 Complete Coefficient Table of Harmonic Regression (Negative Binomial) Model (Pseudo R² = 0.6057)

| Variable | Coefficient | Std. Error | z-value | P-value | 95% CI |
| --- | --- | --- | --- | --- | --- |
| Intercept (const) | -2.9381 | 0.324 | -9.068 | <0.001 | -3.573--2.303 |
| Time Trend (time_idx) | 0.0098 | 0.015 | 0.676 | 0.499 | -0.019-0.038 |
| Seasonal cos Term (cos1) | 1.1039 | 0.237 | 4.657 | <0.001 | 0.639-1.569 |
| Seasonal sin Term (sin1) | -0.8135 | 0.248 | -3.276 | 0.001 | -1.3--0.327 |
| T1 × cos1 (cos1_T1) | 0.3537 | 0.698 | 0.507 | 0.612 | -1.014-1.722 |
| T1 × sin1 (sin1_T1) | 1.558 | 0.562 | 2.773 | 0.006 | 0.457-2.659 |
| T2 × cos1 (cos1_T2) | -0.4289 | 0.729 | -0.588 | 0.556 | -1.858-1 |
| T2 × sin1 (sin1_T2) | -0.2404 | 0.586 | -0.41 | 0.682 | -1.39-0.909 |
| T1 Level Effect (post_T1) | -1.6694 | 0.566 | -2.951 | 0.003 | -2.778--0.561 |
| T2 Level Effect (post_T2) | 1.6028 | 0.535 | 2.999 | 0.003 | 0.555-2.65 |

Table S10 Comparison of key variable effects between Negative Binomial and Quasi-Poisson models for HRSV positive counts

| Variable | Negative Binomial Model (Preferred) | | Quasi-Poisson Model (Dispersion-Adjusted) | |
| --- | --- | --- | --- | --- |
|  | Coefficient (95% CI) | P-value | Coefficient (95% CI) | P-value |
| Overall Time Trend (time_idx) | 0.0358 (0.004–0.067) | 0.025 | 0.0171 (0.014–0.020) | 0.088 |
| NPI Suppression Slope (time_after_T1) | -0.5315 (-0.875–-0.188) | 0.002 | -0.5351 (-0.659–-0.411) | 0.213 |
| NPI Lifting Level Effect (post_T2) | 6.9976 (4.373–9.623) | <0.001 | 6.7963 (5.643–7.950) | 0.089 |
| Seasonal cos Term (cos1) | 1.1150 (0.749–1.481) | <0.001 | 1.2578 (1.213–1.303) | <0.001 |
| Seasonal sin Term (sin1) | -0.6512 (-1.035–-0.267) | <0.001 | -0.5476 (-0.583–-0.512) | <0.001 |
| Model Fit Metrics | Pseudo R²=0.7136; Dispersion=0.714 |  | Pseudo R²=1.000; Dispersion=46.077 |  |

Table S11 Coefficient Comparison Between Full Negative Binomial Model and Dispersion-Adjusted Quasi-Poisson Model

| Variable | Negative Binomial Model | | | | Quasi-Poisson Model (Adjusted) | | | |
| --- | --- | --- | --- | --- | --- | --- | --- | --- |
|  | Coefficient | Std. Error | z-value | P-value | Coefficient | Adjusted Std. Error | z-value | P-value |
| const | -3.4571 | 0.352 | -9.823 | <0.001 | -2.9257 | 0.267 | -10.94 | <0.001 |
| time_idx | 0.0358 | 0.016 | 2.242 | 0.025 | 0.0171 | 0.01 | 1.708 | 0.088 |
| post_T1 | -0.397 | 0.808 | -0.492 | 0.623 | -0.0331 | 0.858 | -0.039 | 0.969 |
| time_after_T1 | -0.5315 | 0.175 | -3.032 | 0.002 | -0.5351 | 0.43 | -1.244 | 0.213 |
| post_T2 | 6.9976 | 1.339 | 5.225 | <0.001 | 6.7963 | 3.995 | 1.701 | 0.089 |
| time_after_T2 | 0.2884 | 0.175 | 1.646 | 0.1 | 0.341 | 0.43 | 0.793 | 0.428 |
| cos1 | 1.115 | 0.187 | 5.969 | <0.001 | 1.2578 | 0.156 | 8.072 | <0.001 |
| sin1 | -0.6512 | 0.196 | -3.326 | <0.001 | -0.5476 | 0.124 | -4.412 | <0.001 |
|  | Dispersion = 0.714 (Pearson χ²/df_resid); Pseudo R² = 0.7136 | | | | Dispersion = 46.077 (Pearson χ²/df_resid); Pseudo R² = 1.000 | | | |

Table S12 Results of multivariate logistic regression analysis for HRSV infection risk factors

| Variable | Coefficient | OR | 95% CI | P-value |
| --- | --- | --- | --- | --- |
| C(age_group, Treatment(reference="[6,18)"))[T.<1] | 2.1992 | 9.0182 | 8.3091-9.7879 | <0.001 |
| C(age_group, Treatment(reference="[6,18)"))[T.[1,3)] | 1.7158 | 5.5613 | 5.1227-6.0375 | <0.001 |
| C(age_group, Treatment(reference="[6,18)"))[T.[3,6)] | 0.8930 | 2.4424 | 2.2469-2.6549 | 0.5416 |
| C(gender)[T.male] | -0.0151 | 0.9850 | 0.9383-1.0340 | <0.001 |
| C(patient category)[T.outpatient] | -0.5259 | 0.5910 | 0.4456-0.7840 | <0.001 |
| month_sin | 1.0708 | 2.9177 | 2.7828-3.0590 | <0.001 |
| month_cos | 1.3819 | 3.9824 | 3.8074-4.1656 | <0.001 |
| T1 | -1.1353 | 0.3213 | 0.2572-0.4014 | <0.001 |
| T2_2023 | 0.8184 | 2.2669 | 2.1284-2.4145 | <0.001 |
| T2_2024 | -0.7084 | 0.4924 | 0.4645-0.5221 | <0.001 |


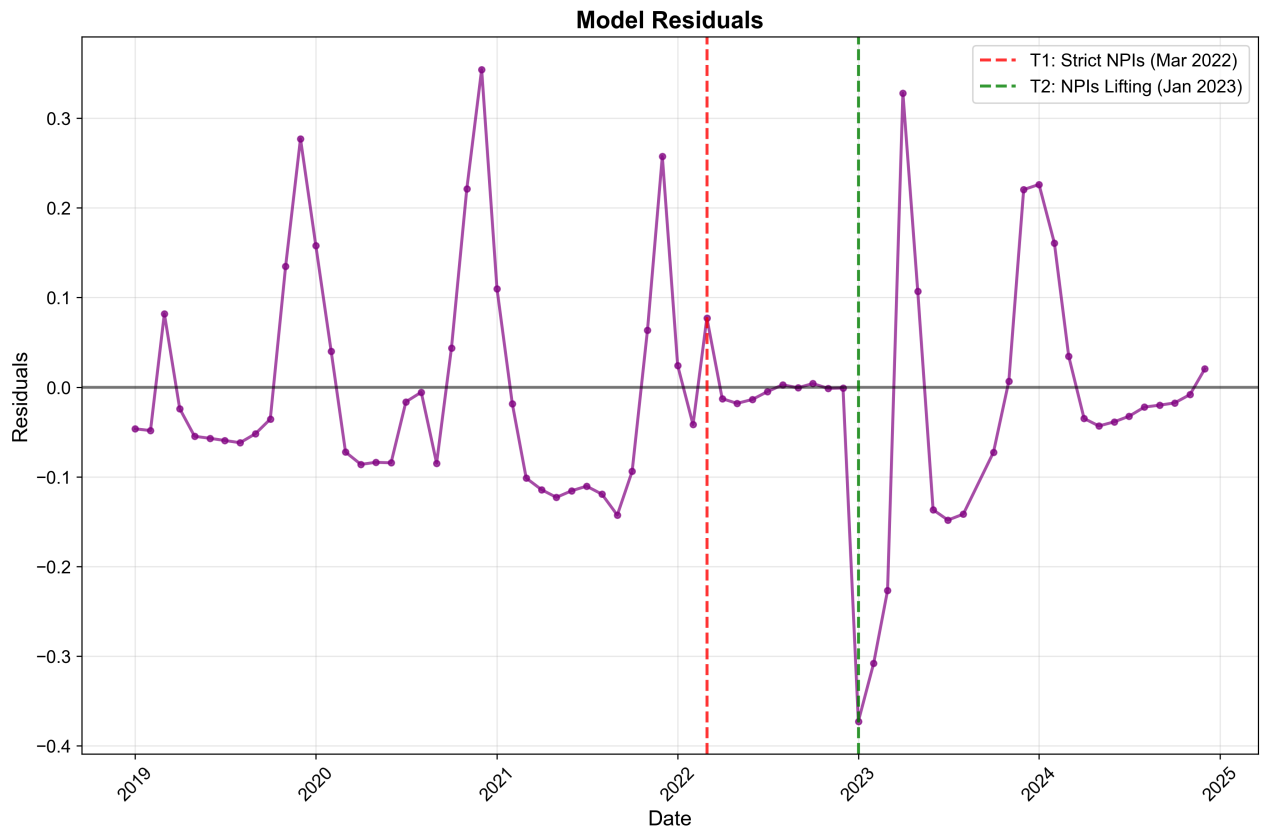


**Fig. S1 Residual time series of the Interrupted Time-Series Analysis (ITSA) model**

This figure shows the residual distribution of the ITSA model (Negative Binomial Generalized Linear Model) over time. Residuals are calculated as the difference between observed HRSV positivity rates and model-fitted values. Vertical dashed lines mark the two key intervention points: T1 (March 2022, strict NPIs implementation) and T2 (January 2023, NPIs lifting). No systematic residual patterns indicate good model fit.


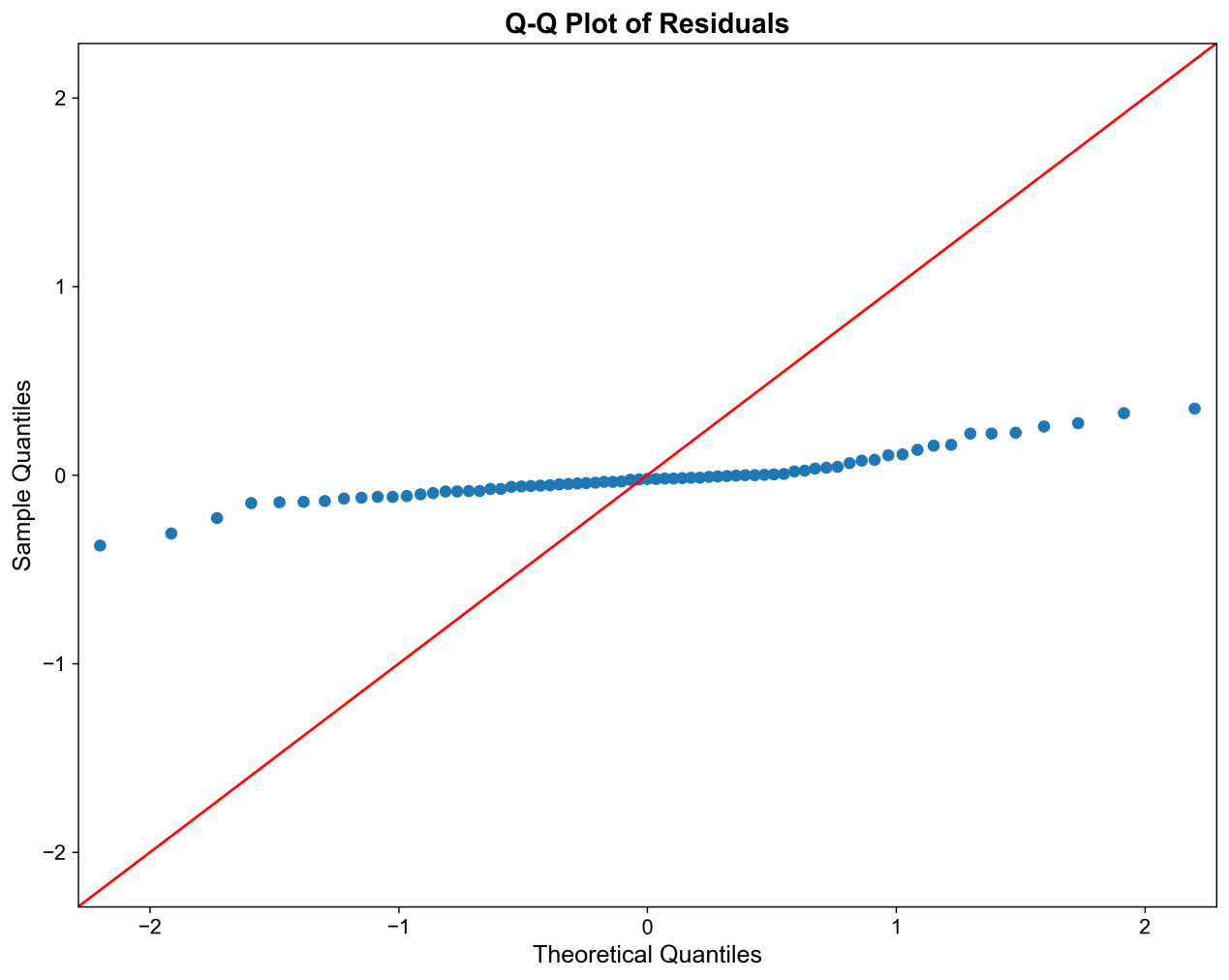


**Fig. S2 Q-Q plot of residuals from the Interrupted Time-Series Analysis (ITSA) model**

This Q-Q plot is used to verify the normality of residuals from the ITSA model. Points approximately following the diagonal line indicate that the residuals conform to a normal distribution, supporting the reliability of the model's statistical inferences.

**
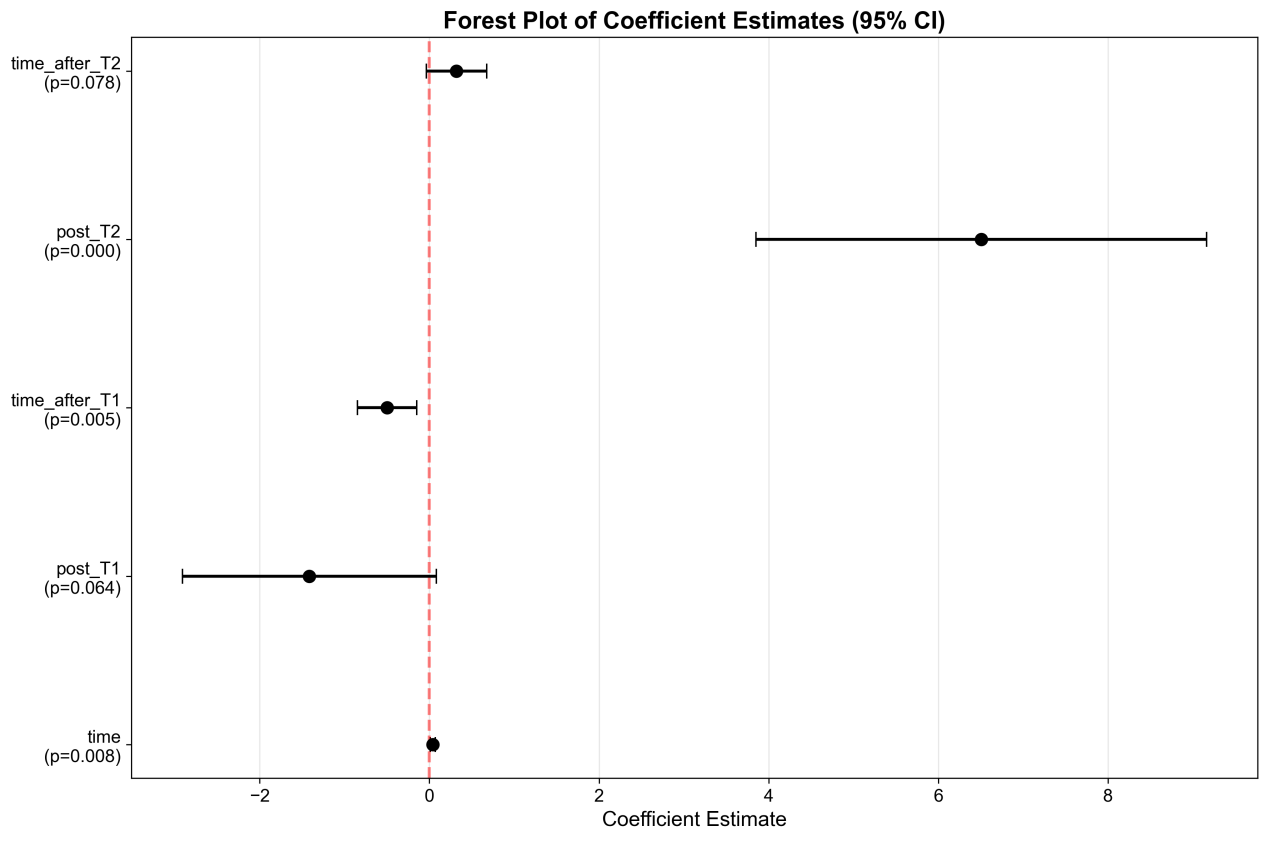
**

**Fig. S3 Forest plot of coefficient estimates from the Interrupted Time-Series Analysis (ITSA) model**

This forest plot displays the coefficient estimates and 95% confidence intervals of each variable in the ITSA model. Variables include intercept, time trend, immediate and trend effects of T1 and T2 interventions. The horizontal line at x=0 indicates no effect; coefficients with confidence intervals not crossing 0 are statistically significant, reflecting the direction and magnitude of each variable's impact on HRSV positivity rates.

**
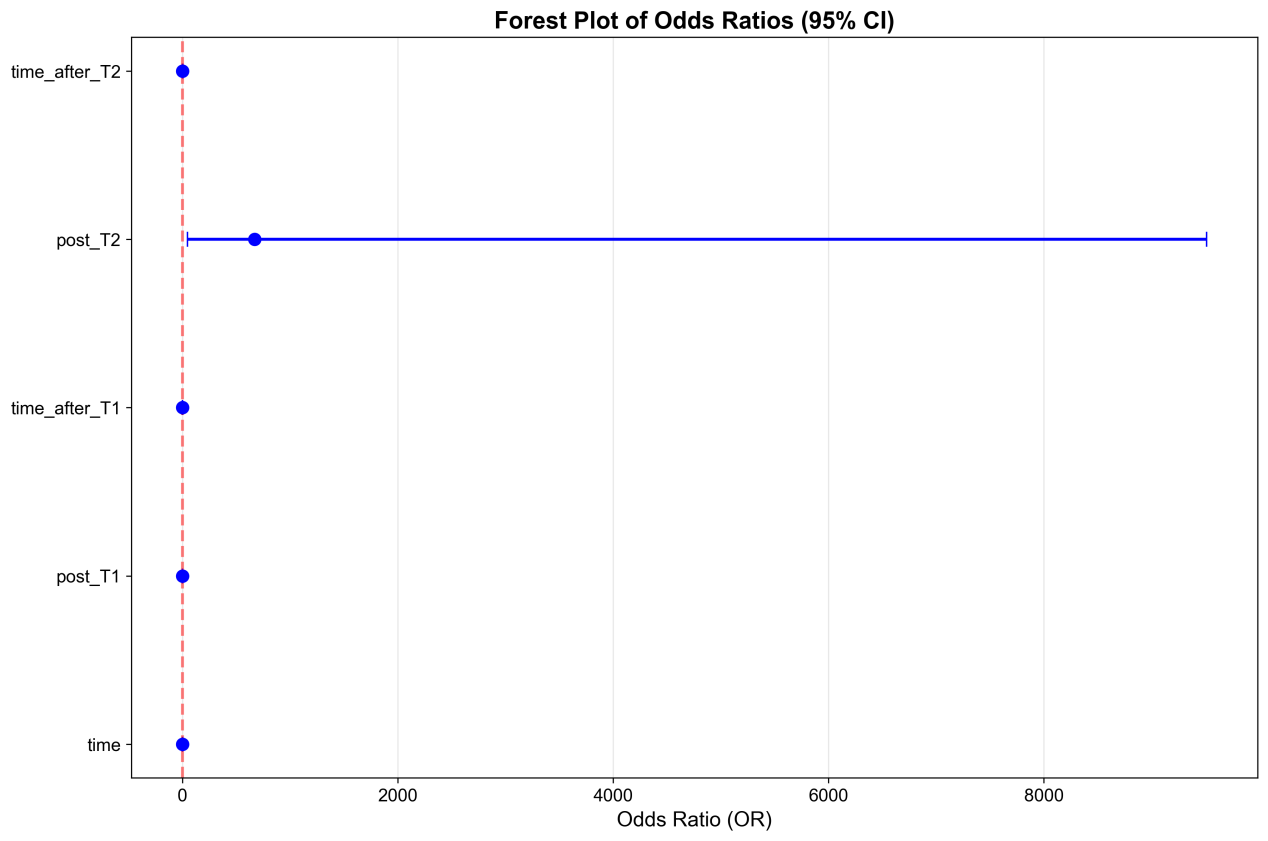
**

**Fig. S4 Forest plot of odds ratios (95% CI) for HRSV infection risk factors across intervention periods**

This forest plot displays the odds ratios (OR) and 95% confidence intervals for variables in the logistic regression model analyzing HRSV infection risk. Variables include:

time: Baseline time trend of HRSV positivity rate.

post_T1: Immediate effect of the first non-pharmaceutical intervention (T1, March 2022).

time_after_T1: Trend effect during the T1 intervention period.

post_T2: Immediate effect of lifting non-pharmaceutical interventions (T2, January 2023).

time_after_T2: Trend effect after T2 intervention.

The vertical dashed line at OR=0 (for time-related variables, interpreted as effect magnitude relative to baseline) and the horizontal axis represents the OR. Confidence intervals not crossing 0 (for time-related) or 1 (for binary interventions, though here presented as OR magnitude) indicate statistical significance. The extremely large OR for post_T2 reflects a strong rebound effect of HRSV positivity rate after lifting NPIs.


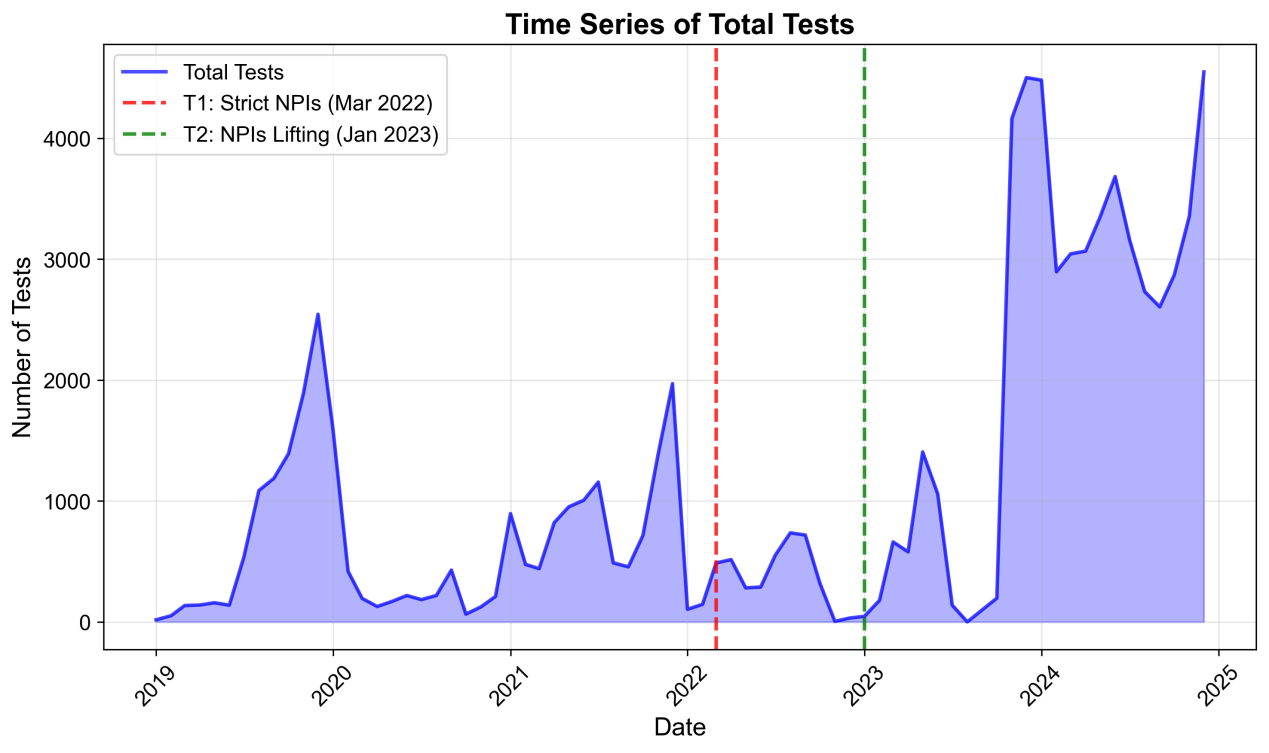


**Fig. S5 Time series of total HRSV testing volume (2019-2024)**

This line chart displays the monthly total number of HRSV tests conducted at Henan Children's Hospital from 2019 to 2024. Vertical dashed lines mark T1 (March 2022) and T2 (January 2023). The testing volume peaked in 2024 (39,793 cases), and no obvious correlation between testing volume and positivity rate was observed, confirming that the epidemiological trends reflect true changes rather than surveillance artifacts.

**
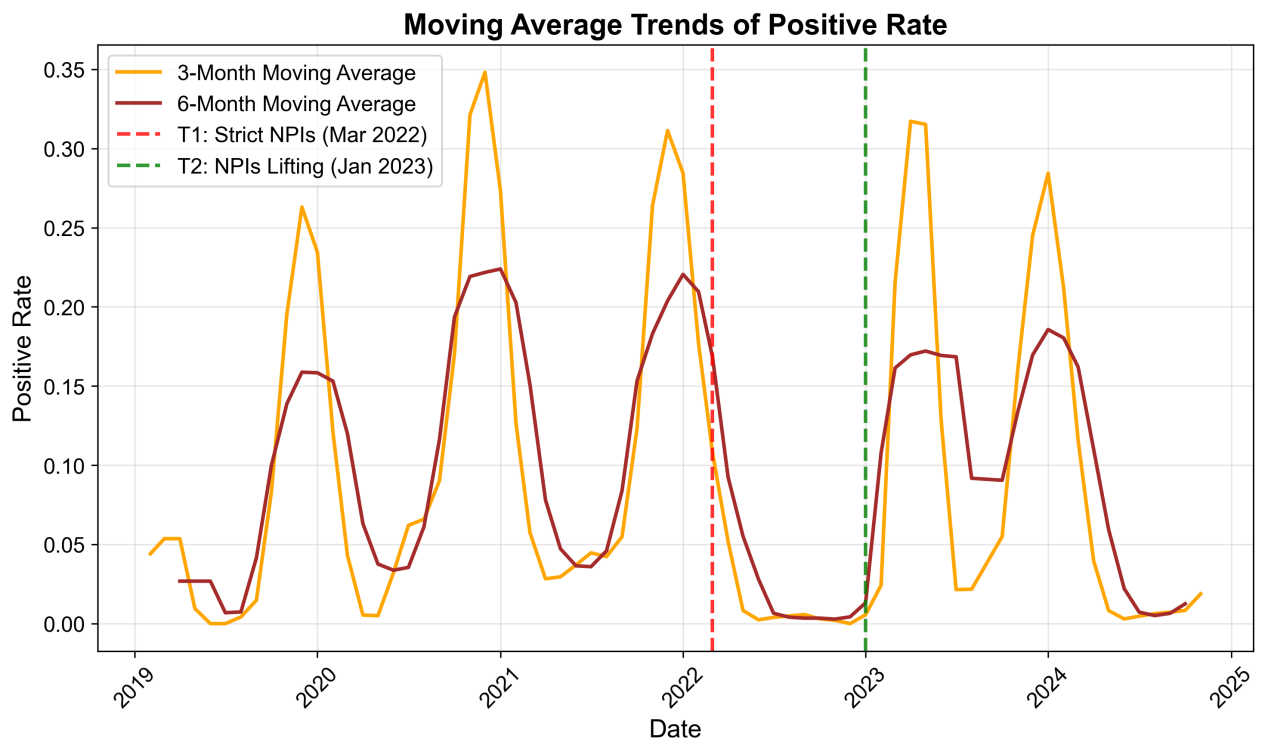
**

**Fig. S6 Moving average trends of HRSV positivity rates (2019-2024)**

This figure shows the 3-month and 6-month moving average trends of HRSV positivity rates. The orange line represents the 3-month moving average, and the brown line represents the 6-month moving average. Vertical dashed lines mark T1 and T2 interventions. The moving average curves clearly reflect the "suppression-rebound-decline" pattern of HRSV positivity rates, consistent with the ITSA model results.


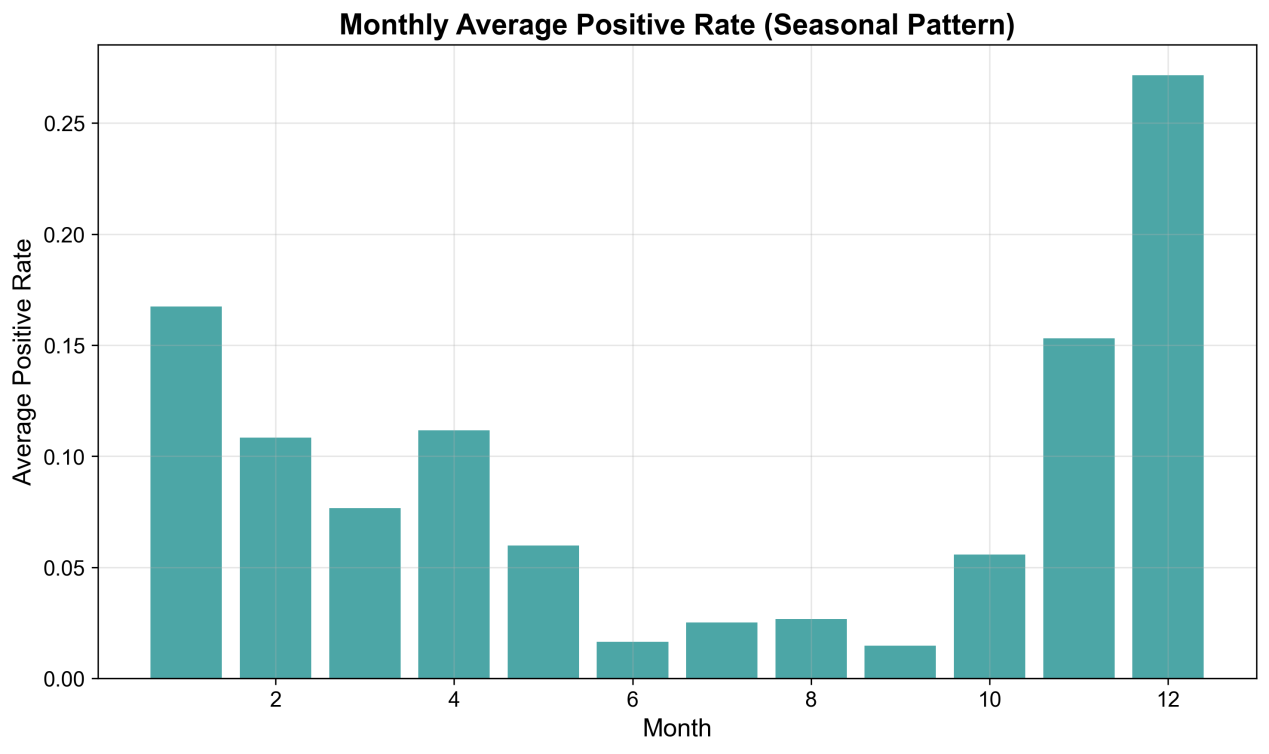


**Fig. S7 Monthly average HRSV positivity rate (seasonal pattern, 2019-2024)**

This bar chart displays the average HRSV positivity rate for each month across the 6-year study period. The horizontal axis represents months (1–12), and the vertical axis represents the average positivity rate. The figure reflects the inherent seasonal characteristics of HRSV infections, with traditional peaks concentrated in winter (November–January) and disrupted patterns during the NPI period.

**
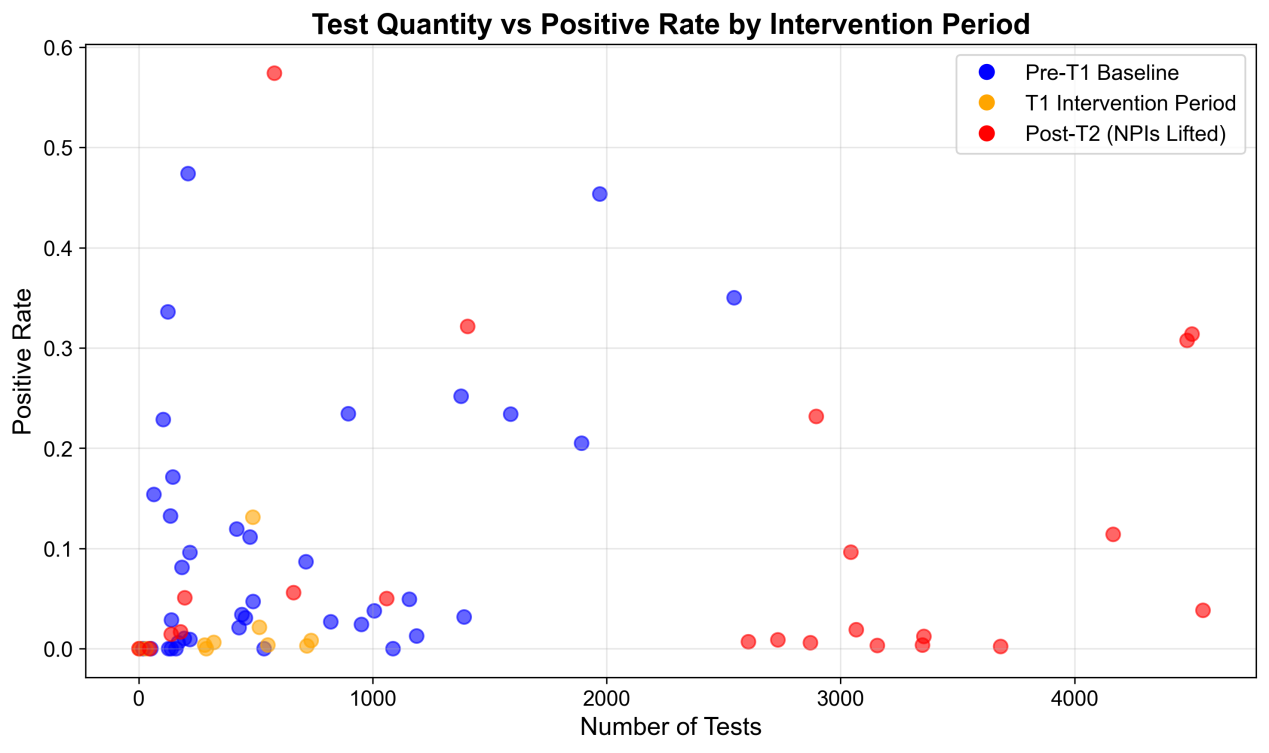
**

**Fig. S8 Scatter plot of HRSV testing volume vs. positivity rate by intervention period**

This scatter plot shows the relationship between monthly HRSV testing volume and positivity rate, with points colored by intervention period (blue: Pre-T1 baseline, orange: T1 intervention period, red: Post-T2 NPIs lifted period). No positive correlation between testing volume and positivity rate was observed, and points are clustered by period, confirming that the observed positivity rate changes are driven by epidemiological factors rather than testing intensity.

**
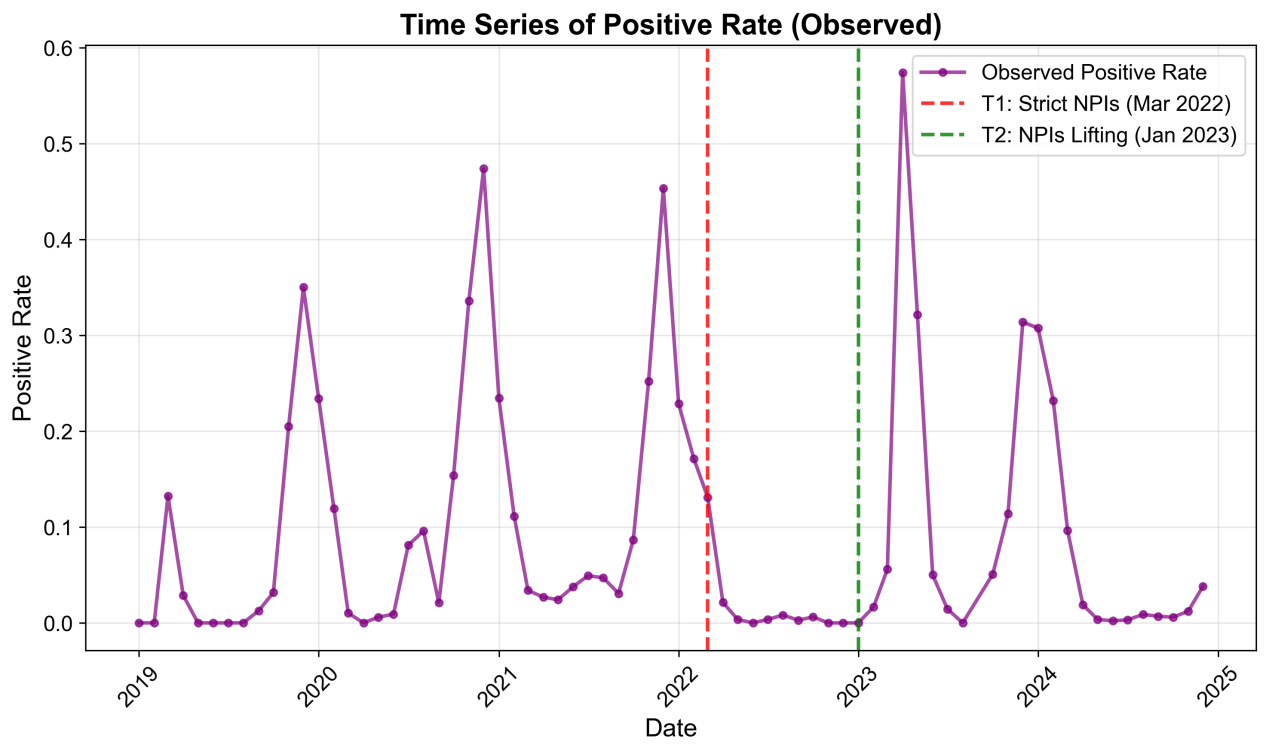
**

**Fig. S9 Time series of observed HRSV positivity rates (2019–2024)**

This line chart displays the monthly observed HRSV positivity rates from 2019 to 2024. Vertical dashed lines mark T1 (March 2022) and T2 (January 2023). The figure clearly shows the sharp decline in positivity rate during the strict NPI period (2022), the surge in 2023 (especially the spring peak in April–May), and the subsequent decline in 2024, reflecting the profound impact of NPIs on HRSV epidemiology.

**
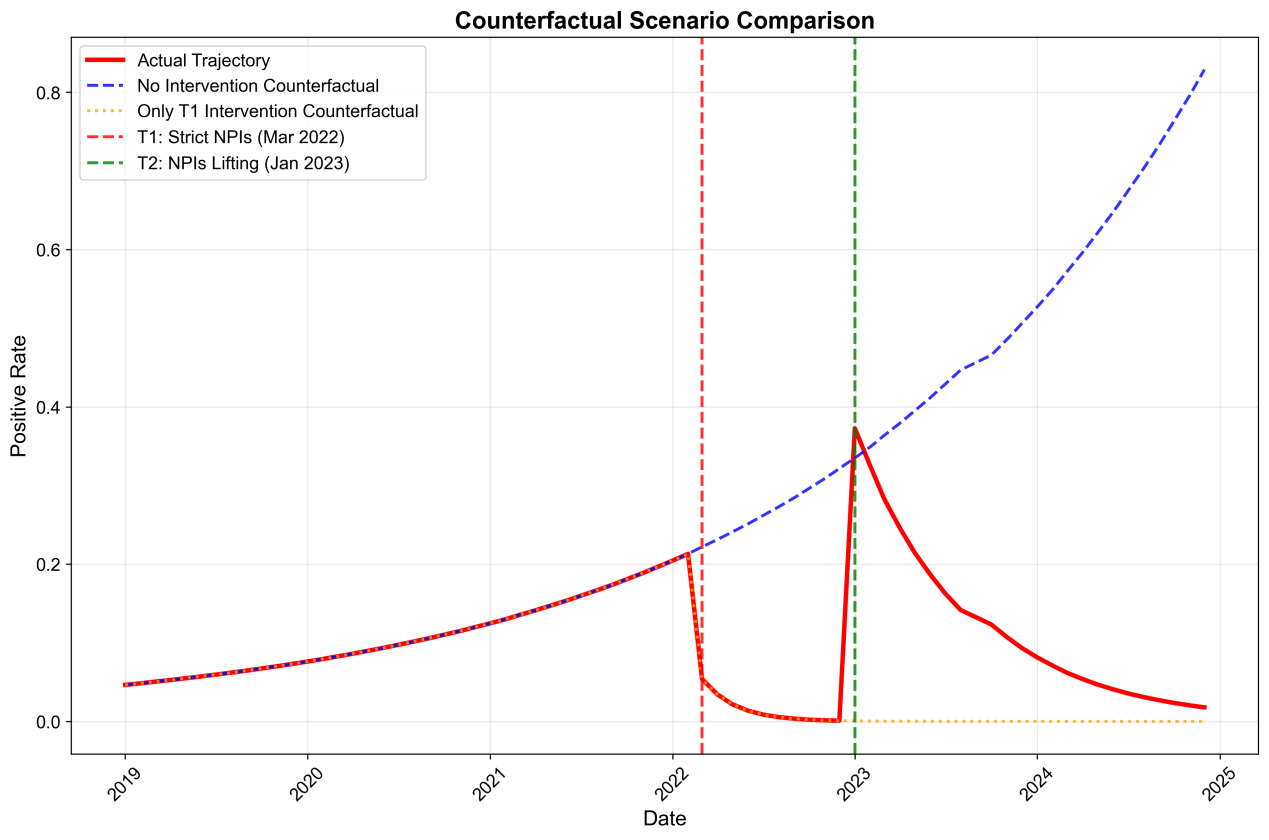
**

**Fig. S10 Counterfactual scenario comparison of HRSV positivity rates**

This figure compares three scenarios: the actual trajectory of HRSV positivity rates (red solid line), the counterfactual scenario without any interventions (blue dashed line), and the counterfactual scenario with only T1 intervention (orange dotted line). Vertical dashed lines mark T1 and T2 interventions. The gap between the actual trajectory and counterfactual scenarios quantifies the net effect of NPI interventions, confirming the suppression effect of T1 and the rebound effect of T2.
